# Evaluation of Clinical and Economic Outcomes Following Implementation of a Medicare Pay-for-Performance Program for Surgical Procedures

**DOI:** 10.1001/jamanetworkopen.2021.21115

**Published:** 2021-08-18

**Authors:** Kyung Mi Kim, Justin S. White, Wendy Max, Susan A. Chapman, Ulrike Muench

**Affiliations:** 1 Clinical Excellence Research Center, Stanford University School of Medicine, Palo Alto, California; 2 Department of Social and Behavioral Sciences, University of California School of Nursing, San Francisco; 3 Philip R. Lee Institute for Health Policy Studies, University of California School of Medicine, San Francisco; 4 Department of Epidemiology & Biostatistics, University of California School of Medicine, San Francisco; 5 Institute for Health & Aging, University of California, San Francisco

## Abstract

**Importance:**

Surgical complications increase hospital costs by approximately $20 000 per admission and extend hospital stays by 9.7 days. Improving surgical care quality and reducing costs is needed for patients undergoing surgery, health care professionals, hospitals, and payers.

**Objective:**

To evaluate the association of the Hospital-Acquired Conditions Present on Admission (HAC-POA) program, a mandated national pay-for-performance program by the Centers for Medicare & Medicaid Services, with surgical care quality and costs.

**Design, Setting, and Participants:**

A cross-sectional study of Medicare inpatient surgical care stays from October 2004 through September 2017 in the US was conducted. The National Inpatient Sample and a propensity score–weighted difference-in-differences analysis of hospital stays with associated primary surgical procedures was used to compare changes in outcomes for the intervention and control procedures before and after HAC-POA program implementation. The sample consisted of 1 317 262 inpatient surgical episodes representing 1 198 665 stays for targeted procedures and 118 597 stays for nontargeted procedures. Analyses were performed between November 1, 2020, and May 7, 2021.

**Exposures:**

Implementation of the HAC-POA program for the intervention procedures included in this study (fiscal year 2009).

**Main Outcomes and Measures:**

Incidence of surgical site infections and deep vein thrombosis, length of stay, in-hospital mortality, and hospital costs. Analyses were adjusted for patient and hospital characteristics and indicators for procedure type, hospital, and year.

**Results:**

In our propensity score–weighted sample, the intervention procedures group comprised 1 047 351 (88.5%) individuals who were White and 742 734 (60.6%) women; mean (SD) age was 75 (6.9) years. The control procedures group included 94 715 (88.0%) individuals who were White, and 65 436 (60.6%) women; mean (SD) age was 75 (7.1) years. After HAC-POA implementation, the incidence of surgical site infections in targeted procedures decreased by 0.3 percentage points (95% CI, −0.5 to −0.1 percentage points; *P* = .02) compared with nontargeted procedures. The program was associated with a reduction in length of stay by 0.5 days (95% CI, −0.6 to −0.4 days; *P* < .001) and hospital costs by 8.1% (95% CI, −10.2% to −6.1%; *P* < .001). No significant changes in deep vein thrombosis incidence and mortality were noted.

**Conclusions and Relevance:**

The findings of this study suggest that the HAC-POA program is associated with small decreases in surgical site infection and length of stay and moderate decreases in hospital costs for patients enrolled in Medicare. Policy makers may consider these findings when evaluating the continuation and expansion of this program for other surgical procedures, and payers may want to consider adopting a similar policy.

## Key Points

**Question:**

What is the association between the Hospital-Acquired Conditions Present on Admission program by the Centers for Medicare & Medicaid Services pay-for-performance program and surgical care quality and costs?

**Findings:**

In this cross-sectional study, the Hospital-Acquired Conditions Present on Admission program was associated with a decreased incidence of surgical site infection (0.3 percentage points) in the targeted procedures and a reduction in length of stay (0.5 days) and hospital costs (8.1%). Deep vein thrombosis and in-hospital mortality did not improve.

**Meaning:**

The findings of this study suggest that the pay-for-performance program was associated with improvement on several dimensions of surgical care, including small reductions in surgical site infection and length of stay, and moderate reductions in hospital costs.

## Teaser

This cross-sectional study examines the outcomes of Medicare patients who underwent surgery before and after implementation of the Hospital-Acquired Conditions Present on Admission program.

## Introduction

Surgical care composes 30% of hospital admissions,^1^ 50% of overall hospital costs,^1^ and 50% of all Medicare spending.^2^ In particular, surgical complications, defined as the adverse and unintended results of surgery,^3^ increase hospital costs by approximately $20 000 per admission^4^ and extend hospital stays by 9.7 days.^5^ Improving surgical care quality and reducing costs are thus necessary for patients, health care professionals, hospitals, and payers.

In 2008, the Centers for Medicare & Medicaid Services (CMS) implemented the Hospital-Acquired Conditions Present on Admission (HAC-POA) program to reduce high-cost and high-volume complications among Medicare patients, and it remains in effect today. This mandatory pay-for-performance (P4P) policy penalizes hospitals by no longer paying for the treatment of preventable complications developed during a patient’s hospitalization. The HAC-POA program targets 14 selected conditions; those directly related to surgery include foreign objects retained after surgery, surgical site infection (SSI) following coronary artery bypass graft, cardiac implantable electronic device, bariatric surgery, certain orthopedic procedures, and deep vein thrombosis (DVT) or pulmonary embolism following certain orthopedic procedures.

Although it has been more than 10 years since the HAC-POA program was implemented, little is known about whether the program is associated with improved surgical care outcomes. Three studies have examined surgical outcomes associated with the HAC-POA program, but issues with study design, such as the absence of a comparison with the prepolicy period,^6^ a control group subject to spillover effects,^7^ and a short study period,^8^ have limited the evaluation of the HAC-POA program.

The aim of this cross-sectional study was to evaluate the association between the HAC-POA program and surgical care quality and costs using a difference-in-differences method. Specifically, we examined surgical complications (ie, SSI and DVT), length of stay (LOS), in-hospital mortality, and hospital costs among Medicare patients who underwent the HAC-POA–targeted surgical procedures compared with nontargeted procedures.

## Methods

### Data and Study Population

We used the National Inpatient Sample of the Healthcare Cost and Utilization Project (HCUP) from October 2004 through September 2017 to examine the association between the HAC-POA program and the incidence of SSI and DVT, LOS, in-hospital mortality, and hospital costs. The unit of analysis was an episode of inpatient surgical care, defined as the hospital stay with its accompanying events associated with the primary surgical procedure. We identified the primary surgical procedure using *International Classification of Diseases, Ninth Revision* (*ICD-9*) and *International Statistical Classification of Diseases, 10th Revision, Clinical Modification* (*ICD-10-CM*) codes (eTable 1 in the Supplement). The study was exempted by the University of California, San Francisco, Institutional Review Board because it was a secondary analysis of deidentified data. The study followed Strengthening the Reporting of Observational Studies in Epidemiology (STROBE) reporting guideline for cross-sectional studies.

**Figure 1.**
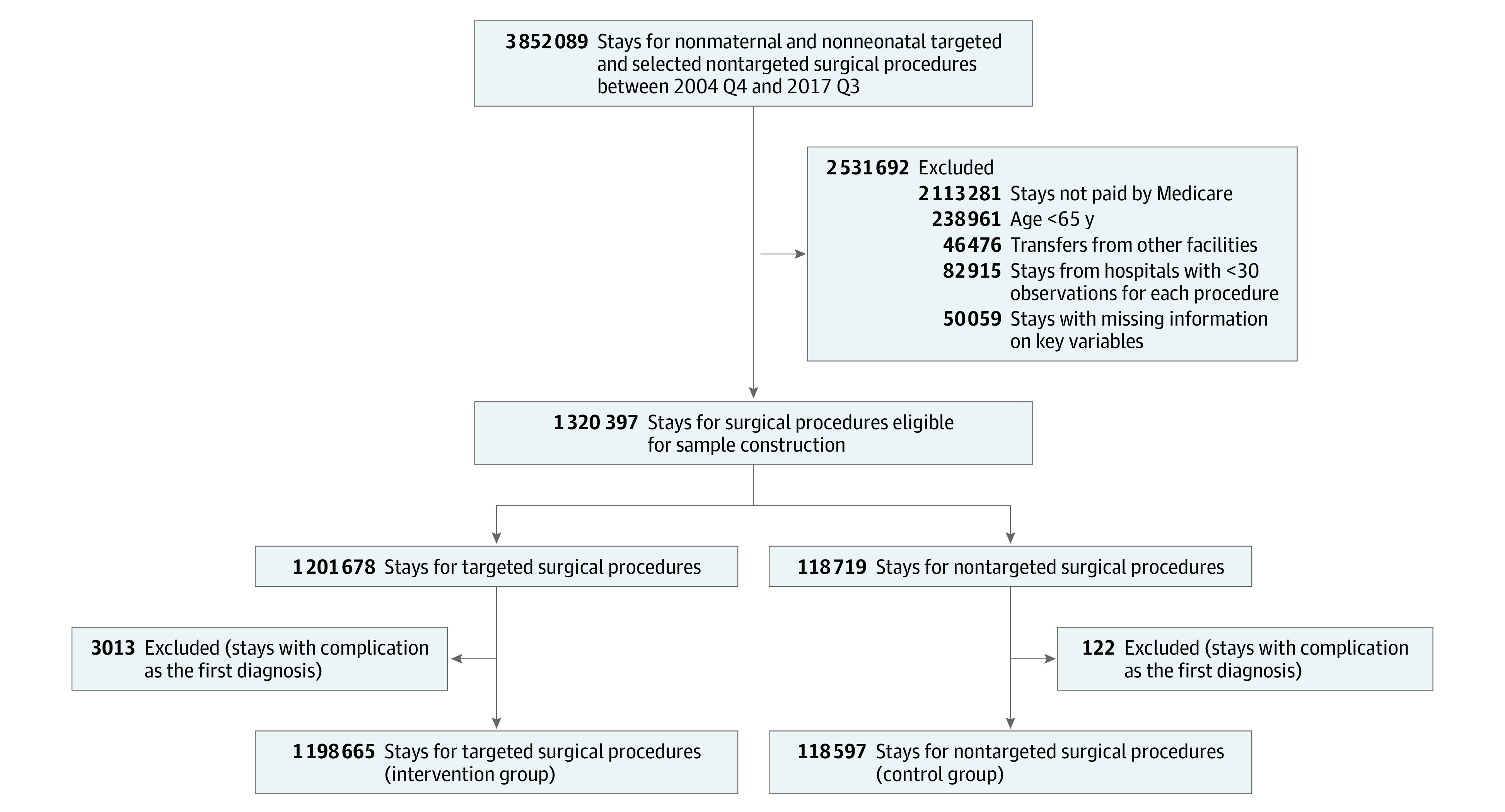
Flow Diagram of Sample Selection Q3 indicates third quarter; Q4, fourth quarter.

We applied several exclusion criteria: (1) maternal or neonatal inpatient services because the risk of complications and treatment management differs in these populations due to pregnancy-related physiologic changes,^9,10,11,12^ (2) transfers from other facilities,^13^ (3) observations with surgical complications as the first diagnosis (potentially preexisting conditions present on admission),^14^ (4) hospitals with fewer than 30 observations for each procedure to avoid unstable estimates due to small sample size,^15^ (5) hospital stays not paid by Medicare and not subject to the HAC-POA program (eg, hospital stays at critical access hospitals),^16^ and (6) observations with missing information on key study variables. Our sample consisted of Medicare-covered inpatient surgical care, representing 1 198 665 hospital stays for targeted procedures and 118 597 stays for nontargeted procedures. Figure 1 shows the sample selection process.

### Targeted vs Nontargeted Procedures and Outcomes

Targeted surgical procedures included in this study were cardiac implantable electronic devices, bariatric surgery, certain orthopedic procedures (ie, spine, shoulder, and elbow), total knee arthroplasty, and total hip arthroplasty. We excluded coronary artery bypass graft from the targeted procedures because coronary artery bypass graft was targeted by other P4P programs (eg, the Premier Hospital Quality Incentive Demonstration), making it difficult to isolate the association with HAC-POA. We selected surgical procedures not targeted by the HAC-POA program (henceforth control procedures) from hospitals that performed both targeted and nontargeted procedures. Outcomes in these procedures indicate what would have happened to the targeted procedures (henceforth intervention procedures) without the policy.^17^ By comparing outcomes between the intervention and control procedures, the influence of potential confounders, such as using electronic health records to enhance the care process and other efforts to improve quality, is removed, as long as the association with targeted and nontargeted procedures is stable over time. The control group was selected from procedures not affected by the CMS policy. We selected laparoscopic cholecystectomy and laparoscopic appendectomy procedures as control groups because these procedures have similar average outcome levels (eTable 2 in the Supplement), involve a different surgical specialty (ie, general surgery), and are not affected by care relating to the targeted procedures. In addition, trends in the outcomes (except length of stay) before policy implementation are similar to those in the intervention procedures, and these procedures are high in volume and have a reasonably large number of complications. Additional information on the rationale for procedure selection, including criteria applied and how each criterion was evaluated, is available in eMethods in the Supplement.

The primary outcomes were the incidence of surgical complications (ie, SSI and DVT), measured dichotomously for each episode using *ICD-9* and *ICD-10-CM *codes (eTable 1 in the Supplement). Secondary outcomes were LOS, in-hospital mortality, and hospital costs. Although these outcomes were not the stated aims of the HAC-POA program, we included them because they have been hypothesized to be downstream consequences of pay-for-performance programs.^18,19,20,21,22^ Length of stay was measured as the number of in-hospital days associated with the procedure. Mortality was defined as in-hospital death recorded for a particular surgical procedure. Hospital costs were measured for an inpatient stay with HCUP’s cost-to-charge ratios at the hospital level. We adjusted for inflation using the personal consumption expenditures health-by-function index,^23^ which is the most appropriate for health expenditures.^24^

### Statistical Analysis

To measure the association between the HAC-POA program and surgical patients’ outcomes, we used a propensity score–weighted difference-in-differences analysis, a statistical technique that compares changes in an outcome for intervention and control procedures before and after the policy implementation (eMethods in the Supplement) to estimate the consequences of the program. Difference-in-differences analysis removes bias from observed and time-invariant unobserved confounders and thus enables us to isolate variation due to the policy.^25,26^ Although the HAC-POA launched in the fourth quarter of 2007 (fiscal year 2008), the payment implication of the HAC-POA program for the intervention procedures included in this study began in the fourth quarter of 2008 (fiscal year 2009).

We began by estimating propensity score weights for 4 groups (prepolicy intervention, postpolicy intervention, prepolicy control, and postpolicy control)^27^ because of substantial prepolicy imbalances in the unweighted sample (eMethods and eTable 3 in the Supplement). We then compared weighted characteristics of the inpatient stays of 3 groups (postpolicy intervention, prepolicy control, and postpolicy control) with the prepolicy intervention group using standardized difference in means (eMethods in the Supplement).^28^ We then measured associations of the program with surgical complications with linear probability models because linear models provide unbiased and consistent estimation with fixed effects.^29,30^ We estimated separate models for each primary outcome (SSI and DVT). To estimate hospital costs, linear regression with a log-transformed cost outcome was used, as used in other studies.^31,32^ Length of stay was analyzed using negative binomial regression to allow for overdispersion. All models controlled for patient and hospital characteristics and indicators for procedure type, year, and hosptial. Patient characteristics included race/ethnicity (White, Black, Hispanic, Asian/Pacific Islander, and other), sex, age, median household income for patient’s zip code by quartile, and 29 indicators from the modified Elixhauser comorbidity index. Race/ethnicity was categorized into 5 groups: White, Black, Hispanic, Asian/Pacific Islander, and other. The Asian and Pacific Islander populations were aggregated into a single category as collected by the HCUP. The individual categorized as Native American, multiracial individual, and other were combined into a single category (“other”) because of the small sample size. Hospital characteristics included bed size (small, medium, and large), ownership (public or private), location and teaching status (rural teaching, rural nonteaching, urban nonteaching, and urban teaching), log-transformed surgical volume for each hospital, and type of admission (elective and nonelective).

To determine whether hospitals may have shifted charges to patients without complications to compensate for potential charge penalties in the surgical complication group, we also estimated costs in linear probability models for stays with and without complications using a 3-way interaction (policy indicator × postpolicy indicator × complication indicator) between the difference-in-differences estimator and the indicator for patients with and without complications (eMethods in the Supplement).

We conducted a series of sensitivity analyses to assess the robustness of results. These analyses included alternative regression specifications, such as using logistic regression to model surgical complications and mortality and a 1-part generalized linear model with γ distribution and log link to model hospital costs.^33,34,35^ We also applied several econometric techniques to validate the difference-in-differences approach, including synthetic control methods,^36^ placebo difference-in-differences models, procedure-specific preintervention indicator variables,^37^ and the use of a different control procedure to address concerns related to wound class incomparability between groups. In addition, we assessed for potential negative consequences (increases in costs and lower quality of care) in the noncomplication group. Additional details on these analyses are provided in eMethods in the Supplement.

All analyses were estimated with robust SEs clustered by hospital to account for patients in the same hospital. We applied survey weights to obtain nationally representative estimates and account for HCUP’s complex survey designs. Statistical significance was assessed with a 2-sided significance level of *P* < .05. Analyses were performed between November 1, 2020, and May 7, 2021 using Stata MP, version 15.1 (StataCorp).

## Results

Our sample included 1 317 262 inpatient surgical episodes representing 1 198 665 stays for intervention procedures and 118 597 stays for control procedures. In our propensity score–weighted sample, the intervention group included 1 047 351 (88.5%) individuals who were White and 742 734 (60.6%) women; the mean (SD) age was 75 (6.9) years. The control group included 94 715 (88.0%) individuals who were White and 65 436 (60.6%) women; the mean (SD) age was 75 (7.1) years. Table 1 reports propensity score–weighted characteristics of hospital stays for the 4 groups, indicating that weighting helped achieve balance in covariates and that the group composition of the intervention and control procedures did not change notably over time.

**Table 1.** Propensity Score–Weighted Characteristics of Hospital Stays for the Intervention and Control Groups^a^

Characteristics	No. (%)				Weighted standardized difference in means		
	Intervention group^b^		Control group^c^		Postpolicy intervention vs prepolicy intervention	Prepolicy control vs prepolicy intervention	Postpolicy control vs prepolicy intervention
	Pre (n = 194 076)	Post (n = 1 004 589)	Pre (n = 29 487)	Post (n = 89 110)			
Sex							
Male	76 496 (39.4)	379 435 (39.5)	12 319 (38.9)	40 842 (39.7)	0.00	−0.01	0.01
Female	117 850 (60.6)	625 154 (60.5)	17 168 (61.1)	48 268 (60.3)	0.00	0.01	−0.01
Race/ethnicity							
White	171 767 (88.5)	875 584 (88.2)	24 170 (88.5)	70 545 (87.7)	−0.01	0.00	−0.02
Black	8211 (4.2)	53 648 (4.3)	1473 (4.3)	5480 (4.3)	0.01	0.01	0.00
Hispanic	7539 (3.9)	40 734 (4.0)	2366 (3.8)	8446 (4.3)	0.01	0.00	0.02
Asian/Pacific Islander	2569 (1.3)	12 042 (1.4)	731 (1.3)	2342 (1.6)	0.00	0.00	0.02
Other^d^	3990 (2.1)	22 581 (2.0)	747 (2.0)	2297 (2.0)	0.00	0.00	0.00
Age, mean (SD), y	75.5 (7.0)	74.7 (6.9)	75.5 (7.1)	75.3 (7.2)	−0.12	0.00	−0.03
Median income quartile							
0-25th percentile	44 437 (22.9)	225 199 (24.9)	7357 (21.9)	22 624 (24.0)	0.05	−0.02	0.03
26-50th percentile	52 642 (27.1)	265 581 (28.2)	7573 (27.0)	22 959 (28.4)	0.02	0.00	0.03
51-75th percentile	50 455 (26.0)	266 422 (25.2)	7389 (26.4)	22 937 (25.8)	−0.02	0.01	0.00
76-100th percentile	46 542 (24.0)	247 387 (21.8)	7168 (24.7)	20 590 (21.8)	−0.05	0.02	−0.05
Elixhauser Comorbidity Index							
AIDS	11 (0.0)	120 (0.0)	<5 (0.0)	29 (0.0)	0.00	0.00	0.00
Alcohol abuse	1288 (0.6)	9942 (0.7)	247 (0.7)	1400 (0.8)	0.00	0.00	0.01
Anemia	27 117 (14.0)	132 889 (14.9)	2867 (13.4)	13 524 (13.5)	0.03	−0.02	−0.01
Rheumatoid arthritis/collagen vascular disease	6021 (3.1)	42 293 (3.0)	716 (3.2)	2613 (3.1)	0.00	0.01	0.00
Chronic blood loss anemia	2856 (1.5)	13 067 (1.6)	113 (1.5)	479 (1.2)	0.01	0.00	−0.03
Congestive heart failure	8957 (4.6)	50 053 (4.7)	2714 (4.9)	10 085 (5.0)	0.00	0.01	0.02
Chronic pulmonary disease	29 339 (15.1)	161 903 (15.2)	5168 (15.6)	16 293 (15.7)	0.00	0.02	0.03
Coagulopathy	3437 (1.8)	31 698 (1.8)	595 (1.9)	4225 (2.0)	0.01	0.01	0.01
Depression	14 395 (7.4)	116 980 (7.5)	1673 (7.5)	7328 (7.1)	0.00	0.00	−0.01
Diabetes, uncomplicated	36 029 (18.6)	194 520 (18.9)	6585 (18.6)	22 683 (18.3)	0.01	0.00	−0.01
Diabetes with chronic complications	2960 (1.5)	31 063 (1.5)	614 (1.6)	3246 (1.5)	0.00	0.00	0.00
Drug abuse	215 (0.1)	3281 (0.1)	35 (0.2)	300 (0.1)	0.00	0.01	0.00
Hypertension	131 892 (68.0)	728 377 (68.0)	19 936 (68.6)	66 067 (67.6)	0.00	0.01	−0.01
Hypothyroidism	29 674 (15.3)	185 548 (15.2)	4015 (15.7)	14 438 (15.6)	0.00	0.01	0.01
Liver disease	895 (0.5)	8452 (0.4)	889 (0.4)	4268 (0.5)	0.00	0.00	0.01
Lymphoma	719 (0.4)	4282 (0.4)	160 (0.4)	679 (0.4)	0.00	0.00	0.00
Fluid and electrolyte disorders	19 783 (10.2)	125 082 (10.7)	5580 (10.3)	25 042 (11.0)	0.02	0.01	0.03
Metastatic cancer	742 (0.4)	4060 (0.4)	259 (0.4)	928 (0.5)	0.00	0.01	0.02
Neurologic disorders	10 202 (5.3)	64 384 (5.3)	1462 (5.5)	5531 (5.6)	0.00	0.01	0.01
Obesity	14 734 (7.6)	149 838 (7.5)	2050 (7.4)	11 168 (7.3)	0.00	−0.01	−0.01
Paralysis	1566 (0.8)	8694 (0.8)	346 (0.9)	1207 (0.9)	0.00	0.01	0.01
Peripheral vascular disorders	7333 (3.8)	39 701 (3.8)	1593 (4.0)	6240 (4.2)	0.00	0.01	0.02
Psychoses	2168 (1.1)	15 553 (1.1)	324 (1.2)	1534 (1.1)	0.00	0.01	0.00
Pulmonary circulation disorders	1842 (0.9)	13 983 (1.0)	407 (1.1)	2419 (1.1)	0.01	0.02	0.01
Kidney failure	8536 (4.4)	85 286 (4.5)	1762 (4.6)	11 123 (5.0)	0.01	0.01	0.03
Solid tumor without metastasis	1747 (0.9)	7794 (0.8)	394 (0.9)	1459 (1.0)	0.00	0.00	0.02
Peptic ulcer disease excluding bleeding	52 (0.0)	1068 (0.0)	28 (0.0)	64 (0.0)	0.00	0.00	0.00
Valvular disease	9749 (5.0)	51 962 (4.9)	2008 (5.4)	6599 (5.0)	−0.01	0.02	0.00
Weight loss	1280 (0.7)	12 370 (0.7)	558 (0.7)	4033 (0.8)	0.01	0.01	0.02
Location and teaching status							
Rural	14 293 (7.4)	88 843 (8.2)	3275 (6.8)	9294 (8.0)	0.03	−0.02	0.02
Urban							
Nonteaching	103 966 (53.7)	396 306 (58.7)	17 296 (52.5)	44 085 (58.8)	0.10	−0.02	0.11
Teaching	75 817 (39.1)	519 440 (33.1)	8916 (40.7)	35 731 (33.2)	−0.12	0.03	−0.12
Ownership							
Government	92 641 (47.7)	88 225 (50.2)	12 879 (51.7)	8569 (47.1)	0.05	0.08	−0.01
Private	101 435 (52.3)	916 364 (49.8)	16 608 (48.3)	80 541 (52.9)	−0.05	−0.08	0.01
Bed size							
Small	25 326 (13.0)	196 437 (11.9)	3079 (13.2)	10 698 (13.1)	−0.03	0.00	0.00
Medium	44 183 (22.8)	265 035 (21.9)	7375 (23.1)	24 708 (23.1)	−0.02	0.01	0.01
Large	124 567 (64.2)	543 117 (66.2)	19 033 (63.7)	53 704 (63.7)	0.04	−0.01	−0.01

Abbreviations: Pre, prepolicy period (before the third quarter of 2008); Post, postpolicy period (after the third quarter of 2008).

^a^ The numbers of observations are unweighted raw numbers. Percentages are propensity-score weighted. Percentage sums may not total 100% owing to rounding.

^b^ An intervention group includes patients who underwent procedures targeted by the HAC-POA policy’s (cardiac [implantable electronic devices], orthopedic [spine, neck, shoulder, elbow, total knee, and total hip replacement], and obesity-related bariatric procedures).

^c^ A control group includes patients who underwent a laparoscopic appendectomy or a laparoscopic cholecystectomy.

^d^ The individual categorized as Native American, multiracial individual, and other were combined into a single convenience category (“other”) because of the small sample size.

Table 2 reports covariate-adjusted propensity score–weighted difference-in-differences estimates for each study outcome (propensity score–weighted but unadjusted results are available in eTable 4 in the Supplement). The estimates—comparing changes in the outcomes from the preprogram to postprogram period between the intervention and control procedures—indicated that the HAC-POA program was associated with a significant reduction in SSI incidences of 0.3 percentage points (95% CI, −0.5 to −0.1 percentage points; *P* = .02). The HAC-POA program was also associated with a significant reduction in LOS of 0.5 days (95% CI, −0.6 to −0.4 days; *P* < .001) and hospital costs (−8.1%; 95% CI, −10.2% to −6.1%; *P* < .001). The HAC-POA program was, however, not associated with a reduction in DVT incidence (0.02 percentage points, 95% CI, −0.1 to 0.2; *P* = .80) and mortality (0.05 percentage points; 95% CI, −0.04 to 0.2; *P* = .30).

**Table 2.** Propensity Score–Weighted Estimates of the Associations Between the HAC-POA Program and Surgical Outcomes^a^

Outcome	Difference in the procedures (95% CI)		Difference-in-differences estimate (95% CI)^d^
	Intervention^b^	Control^c^	
SSI	−0.15 (−0.57 to 0.28)	0.14 (−0.26 to 0.55)	−0.29 (−0.53 to −0.05)
DVT	−0.12 (−0.37 to 0.13)	−0.14 (−0.36 to 0.08)	0.02 (−0.13 to 0.17)
Length of stay	−1.27 (−1.41 to −1.13)	−0.73 (−0.86 to −0.60)	−0.54 (−0.65 to −0.43)
Mortality	0.07 (−0.09 to 0.24)	0.02 (−0.16 to 0.20)	0.05 (−0.05 to 0.15)
Hospital costs	−2.7 (−6.55 to 1.23)	5.49 (1.67 to 9.31)	−8.15 (−10.22 to −6.08)

Abbreviations: DVT, deep vein thrombosis; HAC-POA, hospital-acquired conditions present on admission; SSI, surgical site infection.

^a^ Estimates for SSI, DVT, and mortality are predicted probability changes in percentage points. Estimates for length of stay are changes in days. Estimates for hospital costs are changes in percentage.

^b^ The difference in the intervention procedures is the difference in the average marginal effect of the outcome between the prepolicy and postpolicy implementation period for patients in the intervention procedures (ie, cardiac [implantable electronic devices], orthopedic [spine, neck, shoulder, and elbow], and obesity-related bariatric procedures for SSI; total knee and total hip replacement for DVT; and all of the stated procedures for length of stay, mortality, and hospital costs).

^c^ The difference in the control procedures is the difference in the average marginal effect of the outcome between the prepolicy and postpolicy implementation period among patients in the control procedures (ie, laparoscopic cholecystectomy and laparoscopic appendectomy).

^d^ Difference-in-differences estimate is a propensity score–weighted adjusted differential effect of the policy between the intervention procedures and control procedures before and after the policy implementation. For example, the SSI among patients in the intervention procedures decreased 0.3 percentage points after the policy implementation compared with prepolicy relative to the control procedures, and the difference was significant. Length of stay among patients in the intervention procedures decreased by 0.5 days in the postpolicy period compared with the prepolicy period, relative to the control procedures. Inflation-adjusted hospital costs in the intervention procedures decreased 8% after the policy implementation compared with prepolicy relative to the control procedures.

**Figure 2.**
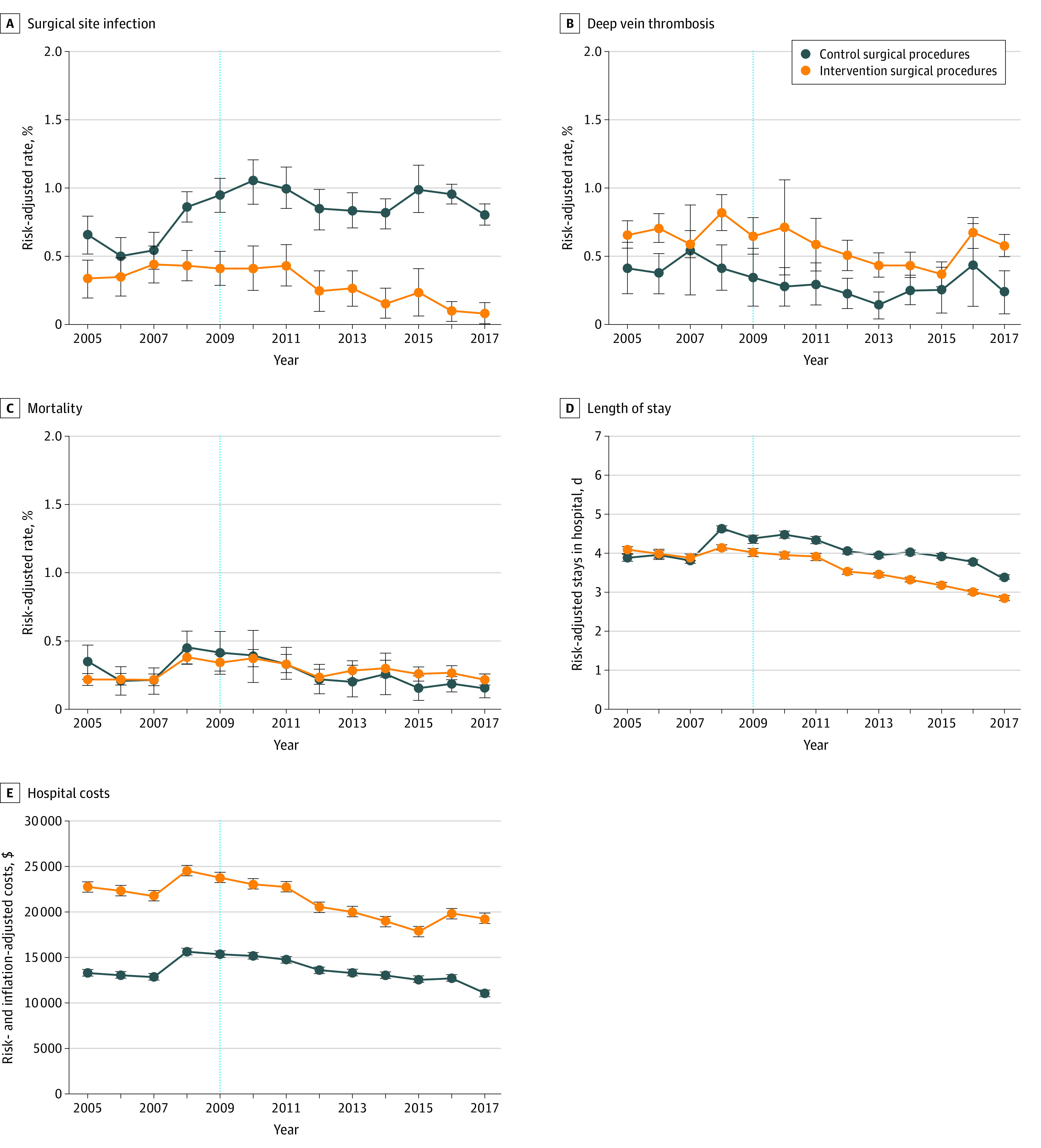
Propensity Score–Weighted Risk-Adjusted Rates of Surgical Complications, Mortality, Length of Stay, and Hospital Costs Before and After the 2009 Implementation of the Pay-for-Performance Program Penalizing Hospital-Acquired Conditions Outcomes for surgical site infection (SSI) (A) and deep vein thrombosis (DVT) (B). The vertical lines indicate the implementation of the Hospital Acquired Conditions Present on Admission (HAC-POA) program. Although the HAC-POA was launched in the fourth quarter of 2007 (fiscal year [FY] 2008), the payment implication of the HAC-POA program for the intervention procedures included in this study began in the fourth quarter of 2008 (FY 2009). Intervention surgical procedures included the HAC-POA policy’s targeted procedures: cardiac (implantable electronic devices), orthopedic (spine, neck, shoulder, and elbow), and obesity-related bariatric procedures for SSI; total knee and total hip replacements for DVT; and all of the stated procedures for length of stay, mortality, and hospital costs. Control procedures include laparoscopic appendectomy and laparoscopic cholecystectomy. The error bars indicate 95% CIs. Models were adjusted for patient characteristics (race/ethnicity, sex, age, median household income for patient’s zip code, and Elixhauser comorbidity index), hospital characteristic (bed size, ownership, location and teaching status, and surgical volume), type of admission, procedure type, and year.

To better understand the outcomes of the policy and validate methodologic assumptions, we graphed outcomes over time (Figure 2). Covariate-adjusted propensity score–weighted incidence rates of SSI improved in the intervention procedures a few years after HAC-POA implementation. In the control procedures, the incidence of SSI worsened in 2008, decreased in 2012, then fluctuated. The incidence of DVT fluctuated in both the intervention and control procedures. The secondary outcomes (LOS, mortality, and hospital costs) worsened in 2008 in both the intervention and control procedures; LOS declined in both the intervention and control procedures starting in 2012, but declined slightly more in the intervention procedures, whereas hospital costs declined in the intervention procedures starting in 2012 but remained flat in the control procedures. The SSI, LOS, and hospital costs graphs show differences in prepolicy trends between the intervention and control procedures. We conducted additional sensitivity analyses to examine this violation in statistical assumption and possible bias in estimates.

### Sensitivity Analyses

Sensitivity analysis showed consistent results across study outcomes, indicating benefits in association with the policy. First, we estimated the likelihood of surgical complications and mortality with average marginal effects using logistic regression and hospital costs with a generalized linear model. The results were nearly identical to those from the linear probability models (eTable 5 in the Supplement). Second, we performed several follow-up analyses to validate the results of the difference-in-differences analyses. To address residual differences in prepolicy trends and assess whether results are associated with other contemporaneous policies, we used synthetic control methods. Results indicated a significant reduction in SSI and no changes in DVT (eFigures 1-4 in the Supplement), consistent with our main results. We also tested for a placebo implementation year of the policy and found no association between the placebo policy variable and surgical outcomes (eTable 6 in the Supplement). To address differences in prepolicy trends, we estimated models with varying prepolicy time trends for procedures and found quantitatively similar results (eTable 7 in the Supplement). Furthermore, using carotid endarterectomy as an alternative control procedure, we observed consistent, but larger, improvements in all outcomes related to the HAC-POA program (eTable 8 in the Supplement). Third, our analysis evaluating potential negative consequences for patients without surgical complications found no significant difference in mortality, LOS, and hospital costs between the intervention and control procedures (eTable 9 in the Supplement).

## Discussion

Our findings of the outcomes of a mandatory national CMS P4P program show significant improvements in several dimensions of surgical care. The incidence of SSI significantly decreased, and we observed a significant reduction in LOS and hospital costs, likely due to the decrease in SSI incidence. However, we did not find evidence of an association between the HAC-POA program and in-hospital mortality. We also found no evidence that the HAC-POA program was associated with cost shifting and lower quality among patients without surgical complications.

Our results are consistent with earlier research reporting that the HAC-POA program was associated with a decreased incidence of SSI.^8^ However, this study also found a statistically significance decrease in DVT. One reason why our results differ might be because previous research adopted a pre-post comparison without a control procedure that did not allow the authors to account for a counterfactual trend. Their study period ranged from 2008 to 2010, leaving later outcomes of the policy unexamined, while we used a difference-in-differences approach with more than 10 years of data.

The magnitude of effect size we found is small. However, cost implications are important, given the substantial costs associated with complications. The cost reduction of 8.1% that we observed in this study is of interest to hospitals, the CMS, and other payers. Considering that the risk-adjusted mean cost for targeted procedures before program implementation was approximately $22 912 per hospital stay, a cost reduction of 8.1% implies a decrease of $1856 for an admission involving a target procedure. Taking into account approximately 1.2 million targeted procedures included in this study, the annual cost savings for hospitals could be $170 million, similar to the cost estimate of complications in the Medicare program suggested by Kandilov and colleagues in 2014.^6^ The reduction in costs observed in this study are related to the decrease in surgical complications leading to fewer additional surgeries, less intensive care use, and shorter length of stay.^38^ Other contributing factors might be changes in practice patterns by health care professionals and hospitals in an effort to improve efficiency of care, including collaborative discharge planning and additional patient education. However, the administration and data collection needs of such a large program could be substantial,^39^ and the costs for hospitals to implement a P4P program should be considered when designing P4P programs.

To date, research evaluating the long-term outcomes of P4P programs is limited, but evidence suggests it may take years for hospitals to change their practices and observe improvements in quality.^19^ In our study, the incidence of SSI did not significantly decrease until 2014—5 years after program implementation. This length of time highlights the importance of evaluating the long-term effects of P4P programs. System and behavioral changes, such as changes to electronic health record infrastructure to improve documentation of presurgical and postsurgical management, and optimizing care coordination between surgeons, nurses, and other specialists, take time.^40,41,42^ Our outcomes are considered a more distal measure of care and, as such, likely require more time than a process measure, such as preoperative β-blockade therapy, which is a proximal measure of care.^43^ Also, the incidence of surgical site infection is only approximately 1% in the intervention procedures, which makes it difficult to improve. Health care professionals and hospitals may disproportionately focus on areas that occur more frequently than SSI. Mortality did not improve even 6 years after HAC-POA program implementation, which is consistent with evidence under the Medicare Premier Hospital Quality Incentive Demonstration^19^ and the Hospital Value-Based Purchasing program.^44^

Previous studies evaluating the long-term outcomes of the Hospital Readmissions Reduction Program, another penalty-based P4P policy, also found improvements in surgical care.^45,46^ Although additional studies are needed to evaluate penalty-based P4P programs, our results provide additional support for the promising benefits of penalty-based P4P programs. Expanding the HAC-POA program to include additional procedures might prove beneficial in improving care for a larger number of surgical patients.

One potential unintended consequence of the HAC-POA program is that hospitals might seek to recover shortfalls by increasing costs to patients without surgical complications. We examined whether patients without surgical complications might have experienced increases in costs but found no such evidence. However, our data show that, for Medicare patients undergoing surgeries for nontargeted procedures, the incidence of SSI increased a year before HAC-POA program implementation and remained high. This increase might suggest that hospitals shifted resources to improve the quality of targeted procedures at the expense of nontargeted procedures.^45^ Alternatively, hospitals and health care professionals may have lacked incentives to improve the quality of nontargeted procedures. Additional research is needed to further explore possible negative consequences of P4P efforts on nontargeted procedures and on specific subgroups of hospitals and patients. For example, hospitals serving a disproportionate share of vulnerable patients may be more likely to be penalized owing to resource limitations. Another challenge for P4P implementation and evaluation is that some hospitals may reject high-risk patients or change coding practices so as not to report complications or claim complications as present-on-admission conditions to avoid penalties.^47,48^

### Limitations

This study has several limitations. First, the observed associations with the implementation of the HAC-POA program may be overestimated owing to undetected or underreported surgical complications. The HCUP data only capture SSIs and DVTs detected during hospitalization and not those occurring after discharge.^12^ However, if rates of missed complications are similar between targeted and nontargeted procedures, it would not bias the result. Second, preintervention trends were significantly different in LOS. Group composition might also have changed over time (eg, characteristics of patients who undergo certain procedure or the composition of clinicians providing care). To address this issue, we used propensity score weighting to remove substantial differences in the composition of each group. We also performed a series of sensitivity analyses, including difference-in-differences analysis with group-specific time trends^37,49^ and analyses using synthetic control methods^36^ to address selection bias across time and across group and confirmed the robustness of our main results. But there remains a potential for confounding from unmeasured time-variant changes. Third, the difference-in-differences model is susceptible to unobserved time-varying confounding, and we cannot rule out confounding from contemporaneous policies implemented during this study period (eg, the Hospital-Acquired Condition Reduction Program) and affected the surgical procedures and outcome measures under study. Fourth, hospital costs related to the implementation of the P4P intervention, such as hiring and training staff to oversee program implementation, could not be assessed in these data. Hospital payments are also specific to the service and payer; thus, hospital-specific cost-to-charge ratios may not fully capture the true costs of specific services. Fifth, although the HCUP, a large-scale data set, has been widely used in health care research, it is reported to have a moderate amount of missing data, especially patient race and ethnicity variables, that may bias the estimates.^50^ To address the concerns about missingness, we used a conditional multiple imputation by chained equation^50^ and found consistent results. Sixth, this study was based on administrative data, which rely on self-reported complications, and thus inherits limitations in coding practice changes. To minimize this limitation, we adjusted for time effects in our modeling, but the potential for confounding from coding practice change remains and the observed association might be overestimated.

## Conclusions

Our study found evidence suggesting improved surgical care related to the implementation of CMS’s national P4P program using a penalty design. Penalties have recently become popular in P4P programs,^51^ likely because health care professionals and hospitals are more responsive to losses than gains.^52^ Although the incidence of surgical complications is low, the costs of complications are high, and our findings suggest that not paying for hospital-acquired infections might successfully encourage hospitals and health care professionals to improve care for surgical patients. Policy makers can use these findings when evaluating the continuation and expansion of this P4P program for the CMS. Other payers also may want to consider implementing similar policies.

## Notes

Supplement. **eMethods.** Detailed Methods **eTable 1.***ICD-9/10-CM* Codes for Defining the Sample **eTable 2.** Surgical Complication Rates for High-Volume Surgical Procedures in the Healthcare Cost and Utilization Project Data FY 2005-2017 **eTable 3.** Propensity Score-Unweighted Characteristics of Hospital Stays for the Pre-Policy Intervention Group, the Post-Policy Intervention Group, the Pre-Policy Control Group, and the Post-Policy Control Group **eTable 4.** Covariate Unadjusted Propensity Score–Weighted Surgical Complication Rates, Length of Stay, and Hospital Costs Before and After the HAC-POA Program Implementation for Hospital Stays for Intervention Procedures and Control Procedures **eTable 5.** Propensity Score–Weighted Estimates of the Association Between the HAC-POA Program and Surgical Site Infection, Deep Vein Thrombosis, and Mortality From the Logistic Regression Model and Hospital Costs From a Generalized Linear Model **eTable 6.** Propensity Score–Weighted Estimates of the Association Between the HAC-POA Program and Surgical Outcomes From Difference-in-Differences Models Using a Placebo Implementation Year **eTable 7.** Propensity Score–Weighted Estimates of the Association Between the HAC-POA Program and Surgical Outcomes From Model Using Intervention and Control Group-Specific Pre-Intervention Time Trends **eTable 8.** Propensity Score–Weighted Estimates of the Association Between the HAC-POA Program and Surgical Outcomes among Patients Who Underwent the Target Surgical Procedures and Patients Who Underwent Carotid Endarterectomy **eTable 9.** Propensity Score–Weighted Estimates of the Association Between the HAC-POA Program and Surgical Outcomes in Patients With Surgical Complications vs. Patients Without Surgical Complications **eFigure 1.** Estimates of the Association Between the HAC-POA Program and Surgical Site Infections: Comparison Between the Intervention Procedures and the Synthetic Control Procedures **eFigure 2.** Estimates of the Association Between the HAC-POA Program and Deep Vein Thrombosis: Comparison Between the Target Procedures and the Synthetic Control Procedures **eFigure 3.** Ratio of Post-policy MSPE and Prepolicy MSPE: Surgical Site Infection **eFigure 4.** Ratio of Post-policy MSPE and Pre-policy MSPE: Deep Vein Thrombosis
eReferences
