# Supplementary material for: Evaluation of Clinical and Economic Outcomes Following Implementation of a Medicare Pay-for-Performance Program for Surgical Procedures
Source: JAMA Netw Open. 2021 Aug 18;4(8):e2121115. doi: 10.1001/jamanetworkopen.2021.21115 (PMC8374611; doi:10.1001/jamanetworkopen.2021.21115)
Supplement: Supplement. — eMethods. Detailed Methods eTable 1.ICD-9/10-CM Codes for Defining the Sample eTable 2. Surgical Complication Rates for High-Volume Surgical Procedures in the Healthcare Cost and Utilization Project Data FY 2005-2017 eTable 3. Propensity Score-Unweighted Characteristics of Hospital Stays for the Pre-Policy Intervention Group, the Post-Policy Intervention Group, the Pre-Policy Control Group, and the Post-Policy Control Group eTable 4. Covariate Unadjusted Propensity Score–Weighted Surgical Complication Rates, Length of Stay, and Hospital Costs Before and After the HAC-POA Program Implementation for Hospital Stays for Intervention Procedures and Control Procedures eTable 5. Propensity Score–Weighted Estimates of the Association Between the HAC-POA Program and Surgical Site Infection, Deep Vein Thrombosis, and Mortality From the Logistic Regression Model and Hospital Costs From a Generalized Linear Model eTable 6. Propensity Score–Weighted Estimates of the Association Between the HAC-POA Program and Surgical Outcomes From Difference-in-Differences Models Using a Placebo Implementation Year eTable 7. Propensity Score–Weighted Estimates of the Association Between the HAC-POA Program and Surgical Outcomes From Model Using Intervention and Control Group-Specific Pre-Intervention Time Trends eTable 8. Propensity Score–Weighted Estimates of the Association Between the HAC-POA Program and Surgical Outcomes among Patients Who Underwent the Target Surgical Procedures and Patients Who Underwent Carotid Endarterectomy eTable 9. Propensity Score–Weighted Estimates of the Association Between the HAC-POA Program and Surgical Outcomes in Patients With Surgical Complications vs. Patients Without Surgical Complications eFigure 1. Estimates of the Association Between the HAC-POA Program and Surgical Site Infections: Comparison Between the Intervention Procedures and the Synthetic Control Procedures eFigure 2. Estimates of the Association Between the HAC-POA Program and Deep Vein Thrombo [file jamanetwopen-e2121115-s001.pdf]

Supplementary Online Content

Kim KM, White JS, Max W, Chapman SA, Muench U. Evaluation of clinical and economic outcomes following implementation of a Medicare pay-for-performance program for surgical procedures. *JAMA Netw Open*. 2021;4(8):e2121115. doi:10.1001/jamanetworkopen.2021.21115

eMethods. Detailed Methods

eTable 1. *ICD-9/10-CM* Codes for Defining the Sample

eTable 2. Surgical Complication Rates for High-Volume Surgical Procedures in the Healthcare Cost and Utilization Project Data FY 2005-2017

eTable 3. Propensity Score-Unweighted Characteristics of Hospital Stays for the Pre-Policy Intervention Group, the Post-Policy Intervention Group, the Pre-Policy Control Group, and the Post-Policy Control Group

eTable 4. Covariate Unadjusted Propensity Score–Weighted Surgical Complication Rates, Length of Stay, and Hospital Costs Before and After the HAC-POA Program Implementation for Hospital Stays for Intervention Procedures and Control Procedures

eTable 5. Propensity Score–Weighted Estimates of the Association Between the HAC-POA Program and Surgical Site Infection, Deep Vein Thrombosis, and Mortality From the Logistic Regression Model and Hospital Costs From a Generalized Linear Model

eTable 6. Propensity Score–Weighted Estimates of the Association Between the HAC-POA Program and Surgical Outcomes From Difference-in-Differences Models Using a Placebo Implementation Year

eTable 7. Propensity Score–Weighted Estimates of the Association Between the HAC-POA Program and Surgical Outcomes From Model Using Intervention and Control Group-Specific Pre-Intervention Time Trends

eTable 8. Propensity Score–Weighted Estimates of the Association Between the HAC-POA Program and Surgical Outcomes Among Patients Who Underwent the Target Surgical Procedures and Patients Who Underwent Carotid Endarterectomy

eTable 9. Propensity Score–Weighted Estimates of the Association Between the HAC-POA Program and Surgical Outcomes in Patients With Surgical Complications vs. Patients Without Surgical Complications

eFigure 1. Estimates of the Association Between the HAC-POA Program and Surgical Site Infections: Comparison Between the Intervention Procedures and the Synthetic Control Procedures

eFigure 2. Estimates of the Association Between the HAC-POA Program and Deep Vein Thrombosis: Comparison Between the Target Procedures and the Synthetic Control Procedures

eFigure 3. Ratio of Post-policy MSPE and Pr-epolicy MSPE: Surgical Site Infection

eFigure 4. Ratio of Post-policy MSPE and Pre-policy MSPE: Deep Vein Thrombosis

eReferences

This supplementary material has been provided by the authors to give readers additional information about their work.

© 2021 Kim KM et al. *JAMA Network Open.*

eMethods. Detailed Methods

Selection of control procedures

Following the literature,[1] we selected nontargeted surgical procedures for a control group based on the following criteria: 1) procedures have similar complication rates on average; 2) procedures are not subject to spillover effects from the intervention procedures; 3) trends in outcomes prior to the policy implementation are parallel between the intervention and control procedures which is a key requirement of our statistical approach; and 4) procedural volume and infection rates are sufficiently large to ensure statistical robustness and account for potential unobserved motivation of providers and hospitals to improve quality of care. To this end, we selected laparoscopic appendectomy and laparoscopic cholecystectomy as control procedures.

We believe that laparoscopic appendectomy and laparoscopic cholecystectomy make the most suitable control group for the following reasons:
1) Laparoscopic appendectomy and laparoscopic cholecystectomy has similar complication rates compared to the intervention procedures (Criterion 1).
2) Laparoscopic appendectomy and laparoscopic cholecystectomy are different surgical specialties (i.e., general surgery) in comparison to the intervention procedures and are thus not likely to be affected by care from intervention procedures (less subject to spillovers) (criterion 2).
3) Our analysis shows parallel trends (Criterion 3) for the majority of outcomes, with the exception length of stay.
4) These procedures are high in volume and sufficiently large complication sample size (Criterion 4).

One might question whether it is reasonable to compare infection rates from clean procedures to clean contaminated procedures. The difference-in-differences method eliminates unobserved time-invariant biases by comparing changes over time between two groups (details of the difference-in-differences method are in Supplement eStudy Methods page 6). Assuming that bacterial load remains consistent in each group over time, the effects of bacterial load would be eliminated by subtracting the between group differences during the pre-policy period from the between group differences during the post-policy period. Nevertheless, to address concerns related to wound class incomparability between groups, we ran sensitivity analyses with different control procedures, including carotid endarterectomy and synthetic control methods.

Control procedure selection criteria checklist

1. In this cross-sectional study, we used a difference-in-differences strategy, which allowed us to compare the changes in outcomes between the intervention and control groups' characteristics that might not have been equivalent. We used laparoscopic appendectomy and laparoscopic cholecystectomy as control procedures. Control procedures have different bacterial compositions than intervention procedures do. Thus, the surgical site infection rate is expected to be worse. In fact, infection rates are higher in the control procedures (Criterion 1). If this were an experimental study with a random assignment of the policy, these two groups might not be the best comparisons for each other due to the procedures' unequal characteristics.

© 2021 Kim KM et al. *JAMA Network Open.*

2. The difference-in-differences technique removes unobserved time-invariant biases by comparing the changes over time between two groups. Bacterial load, a risk factor for infection, is not the same in the intervention and control procedures. Thus, the observed infection rate differs. Because bacterial load was not observed in our data, we could not account for it in our model estimates. However, assuming that bacterial load stays consistent in both the intervention and control groups, the effects of bacterial load would be removed in our analysis since the difference-in differences technique subtracts the between group differences during the pre-policy period from the between group differences during the post-policy period.

3. The difference-in-differences model relies on the assumption that: (1) the policy's effect does not spill over from intervention group to the control group (Criterion 2), (2) the trend in observed outcome differences in the intervention and control procedures is consistent prior to policy implementation (Criterion 3), and (3) consistent composition of groups does not change before and after the policy implementation.

5. We limited the control procedure pool to the top 100 high-volume procedures with sufficiently large complication rates to avoid unstable estimates due to small sample size and to account for unobserved motivational changes (Criterion 4).

6. We consulted existing literature in the selection and evaluation of candidate control procedures. We evaluated four groups of procedures as candidate control procedures: total shoulder replacement, non-Medicare who patients underwent the same procedures, carotid endarterectomy, and laparoscopic appendectomy and laparoscopic cholecystectomy (excluded open appendectomy and open cholecystectomy).

(1) Total shoulder replacement: This was a control procedure used in Kwong et al. (2017)[2]
(2) Non-Medicare patients who underwent the same procedures: This was a control procedure used in Kwong et al. (2017)[2] and Gidwani and Bhattacharya (2015)[3].
(3) Carotid endarterectomy: This is another high-volume procedure that is in the clean wound class.
(4) Laparoscopic appendectomy and cholecystectomy, excluding open appendectomy and cholecystectomy: These are control procedures that have similar complication rates (eTable 2).

<u>Group (1): Total Shoulder Replacement in Kwong et al. (2017)</u>
We considered total shoulder replacement as a control group procedure because of its similarity in terms of service line, having the same wound class and similar levels of microbiome at the surgical site, similar complication rates (Criterion 1), high volume (Criterion 4), and use as a control group in a study by Kwong et al. (2017).[2] We hypothesized that procedures in similar parts of the body could be affected by spillover effects from the policy (Criterion 2), and we tested whether the control group was associated with spillover effects in line with the study by Ryan et al.[4] After careful evaluation, we opted not to use this group because it is subject to spillover (Criterion 2) and violated the parallel trend assumption (Criterion 3). One of the targeted procedures is arthrodesis of the shoulder. For example, a surgeon who performs arthrodesis of the shoulder (the intervention procedure) is also likely to perform total shoulder replacement (the control procedure). Some aspects, such as the surgeon's motivation and the care plan, might influence clinical care in the procedures unexposed to the policy. We statistically

© 2021 Kim KM et al. *JAMA Network Open.*

tested the parallel trend assumption and found that it is violated and also found that it is associated with spillover effect. We concluded that total shoulder replacement is not appropriate for use as a control group procedure.

Group (2): Non-Medicare Patients Who Underwent the Same Procedures in Kwong et al. (2017)[2] and Gidwani and Bhattacharya (2015)[3]
We have concerns with using non-Medicare patients as a control group because a patient's care plan is not payer specific, and other payers implemented similar programs followed by the CMS. Congruent with another study[5]our test also showed evidence of spillover from the intervention group to non-Medicare patients who underwent the same procedures. We opted not to use this group due to these spillover concerns (Criterion 2).

Group (3): Carotid Endarterectomy
We selected this group because it is in the same wound class, it is high in volume (Criterion 4), and it involves a procedure from a different service line. Thus, it is less subject to spillover (Criterion 2). We conducted the same main analyses using this group as a control and had consistent results. We opted not to change this group to the control group in the manuscript because it has more violations in statistical assumptions than our current control group. The parallel trend assumption is not met (Criterion 3), but it was not associated with spillover effects.

Group (4): Laparoscopic Appendectomy and Cholecystectomy (Excluding Open Appendectomy and Cholecystectomy)
We selected this group as control procedures because these procedures are (1) control procedures that have similar complication rates as the intervention procedures, (2) less likely to spillover because they involve different surgical specialties from the intervention procedures, (3) different surgical specialties (i.e., general surgery) in comparison to the intervention procedures and are thus not likely to be affected by care from intervention procedures (less subject to spillovers), and (4) high in volume and yield a sufficiently large complication sample size (Criterion 4). We excluded open appendectomy and cholecystectomy procedures in response to the comments and concerns about comparing procedures with different complication rates (Criterion 1).

7. To account for differences in procedure effects in the outcomes, we included the procedure indicator variables (procedure fixed effect) in the model estimate.

8. As we reported, we were limited in our ability to assess the robustness of our estimates because trends in the two groups were inconsistent before the policy implementation (Criterion 3). To address this issue, we augmented the standard difference-in-differences methodology using four-group propensity score matching. This minimized the differences in the intervention and control groups (the standardized difference in means was < 0.25 in all patient demographic variables and hospital characteristics). To assess the robustness of our results, we constructed the synthetic control procedures to match the levels of outcomes and covariates, indicating an excellent pre-policy fit in the outcomes. However, we did not present this as part of the main analysis because the synthetic control method uses hospital-level difference-in-differences estimation, whereas our main analysis was at the patient-level. Patient-level specification has an advantage in that it controls for individual- and hospital-level heterogeneity across the intervention and control procedures.[4]

© 2021 Kim KM et al. *JAMA Network Open.*

Control procedure selection criteria checklist summary table

The table below summarizes the criteria we used to select the control procedures for the difference-in-differences method and the evaluation of each criterion.

Candidate Control Group	Mean Outcome Levels (control procedures vs. intervention procedures)		Parallel Trend	Spillover
	SSI (intervention group SSI rate = 0.4%)	DVT (intervention group DVT rate = 0.7%)		
Laparoscopic Appendectomy and Laparoscopic Cholecystectomy*	0.9%	0.4%	Met (except LOS)	Met
Open and Laparoscopic Appendectomy and Open and Laparoscopic Cholecystectomy	2.1%	0.6%	Violated	Met
Carotid endarterectomy	3.3%	0.1%	Violated	Met
Total shoulder replacement	1.0%	0.2%	Violated	Violated
Non-Medicare patients underwent the same procedures	0.2%	0.6%	Met	Violated

* We selected and used laparoscopic appendectomy and laparoscopic cholecystectomy as a control group.

Difference-in-differences models

Difference-in-differences is an econometric method to overcome issues with the selection on unobservables. Difference-in-differences has been widely used in the evaluation of health care policies.[4] Policy programs rarely select individuals at random. Instead, such programs purposefully select the target procedures.[6] Thus, the target procedures may have different characteristics compared with the non-target procedures. The basic idea of difference-in-differences is that outcomes are measured for the intervention procedures (the target procedures) and the control procedures (the procedures not exposed to the intervention) before and after the intervention. Any difference between the two groups in the pre-intervention period and the post-intervention period is calculated and defined as a difference-in-differences.[4] Difference-in-differences removes the biases (1) from the permanent difference between the intervention and control procedures due to the omitted variables, thus unobservables, and (2) from differences that could have resulted from trends,[7] if the trends in outcome changes for the intervention and control procedures are similar over the two time periods in the absence of the intervention.[7] Whether the assumption of difference-in-differences is violated can be confirmed by testing the significance of the interaction term between the linear time trend and the intervention procedures during the pre-P4P policy period.[4]

© 2021 Kim KM et al. *JAMA Network Open.*

Applying difference-in-differences in the context of this study, we specified the difference-in-differences model at the patient level as follows:

$$Outcomes_{ikjt} = \beta_0 + \ \beta_1 Treat_k + \ \beta_2 Post_t + \beta_3 Treat_k * Post_t \ + \beta_4 P_{ikjt} + \beta_5 Z_{jt} + \beta_6 H_j + +\beta_7 I_{ijt} + \beta_8 Year + u_{ikjt}$$

where $Outcomes_{ikjt}$for patient i in hospital j receiving procedure k at time t. $Treat$ is equal to 1 if the procedure is targeted by the CMS P4P, Post is equal to 1 for after the third quarter of 2008, P is a vector of patient characteristics, Z is a vector of hospital characteristics, and H, I, and Year are vectors of hospital, procedure and year fixed effects, respectively.
The interaction between P4P and Post is the difference-in-differences estimator. If $\beta_3$has a negative sign, this would indicate that the CMS P4P has improved surgical outcomes (decreased incidence of complications, mortality, length of stay, and hospital costs).

Difference-in-difference-in-differences models

We specified our difference-in-difference-in-differences model by entering a triple interaction term – Treat * Post * Complication – and estimated the model using the following equation to examine whether the policy has had different effects in certain subgroups:

$$Outcomes_{ikjt} = \beta_0 + \ \beta_1 Treat_k + \ \beta_2 Post_t + \beta_3 Complication_{ikjt} + \beta_4 Treat_k \times Post_t \ + \beta_5 Post_t \times Complication_{ijkt} + \beta_6 Treat_k \times Complication_{ijkt} + \beta_7 \ Treat_k \times Post_t \times Complication_{ijkt} + \beta_8 P_{ikjt} + \beta_9 Z_{jt} + \beta_{10} H_j + +\beta_{11} I_{ijt} + \beta_{12} Year + u_{ikjt}$$

In this equation, coefficient β_7 is a difference-in-difference-in-differences estimator for patients with surgical complications. The following table shows how the difference-in-difference-in-differences estimator can be calculated.

	Complication	Before P4P	After P4P	Difference
Target	Complication	$O_{target,pre,complication}$ = B	$O_{target,post,complication}$ = A	$A - B$
NonTarget	Complication	$O_{nontarget,pre,complicat}$ = D	$O_{nontarget,post,complica}$ = C	$C - D$
Difference-in-differences				(A−B) − (C−D)
Target	No Complication	$O_{target,pre,no\ complicati}$ = F	$O_{target,post,no\ complicat}$ = E	$E - F$
NonTarget	No Complication	$O_{nontarget,pre,no\ compli}$ = H	$O_{nontarget,post,no\ compl}$ = G	$G - H$
Difference-in-differences				(E−F) − (G−H)
Difference-in-difference-in-differences				[(A−B) − (C−D)] – [(E−F) − (G−H)]

© 2021 Kim KM et al. *JAMA Network Open.*

Using propensity score in difference-in-differences

We used propensity score weighting based on a study that suggested a specific matching method for the difference-in-differences design,[8] to address the issue that the parallel trend assumption was violated for all outcomes except deep vein thrombosis. We applied propensity score weights to each of the following four groups – pre-policy intervention procedures, post-policy intervention procedures, pre-policy control procedures, and post-policy control procedures – then performed a difference-in-differences analysis. The following steps describe how we integrate propensity scores into a difference-in-differences model.

First, we defined four groups:

- Group 1 –Intervention surgical procedures in the pre-policy period
- Group 2 –Intervention surgical procedures in the post-policy period
- Group 3 –Control surgical procedures in the pre-policy period
- Group 4 –Control surgical procedures in the post-policy period

Second, we estimated the propensity score by regressing a group as a function of patient and hospital characteristics using a multinomial logistic regression. As a result, each observation in our sample has four propensity scores – probability of being in Groups 1, 2, 3, and 4.

Third, we created the weight for each individual using the following formula:

$$\frac{\text{a propensity score of being in Group 1}}{\text{a propensity score of being in the group in which they actually were}}$$

By doing so, observations that were actually in Group 1 have the weights equal to 1, and other observations have weights that represent the similarity to Group 1.

Fourth, we applied the weights and estimated a difference-in-differences model.

We chose this matching method because 1) a particular concern about applying matching in difference-in-differences models exists (there are two elements to consider in a difference-in-difference model: the intervention status and time), 2) the application of this method is appropriate with cross-section data, and 3) this matching method generates fewer covariates with the standardized difference in means greater than 0.25 (represents a substantial difference).[8,9] We tested the balance of covariates the standardized mean difference, which is defined as the difference in means divided by the standard deviation.[8] As shown in eTable 2, there were substantial differences in covariates before applying the propensity score weights, especially between the intervention procedures and the control procedures. However, the propensity score weighting reduced the standardized difference in means to less than 0.25 in all the patient demographic variables and hospital characteristics (Table 1). To examine the sensitivity of our results from the propensity score weighted difference-in-differences analyses, we also performed difference-in-differences analyses with a matched sample using one-to-one matching without replacement, calipers of 0.02 (calculated by 0.25 * standard deviation of propensity score),[10] and enforcing common support. The results were identical, with a slightly larger effect (the results are not presented).

© 2021 Kim KM et al. *JAMA Network Open.*

Sensitivity analyses

We performed a series of sensitivity analyses to assess the robustness of results across model specifications. First, we used the logistic regression to model surgical complications and mortality (eTable 5). Second, we used a one-part generalized linear model (GLM) with gamma distribution and log link to test the robustness of results from the hospital cost models. A Box-Cox approach test and modified Park tests were performed to assure the use of appropriate link and distribution family for a one-part GLM model, respectively.[11,12] Third, we investigated the association between the HAC-POA policy and the incidence of SSI and DVT using a synthetic control method and found consistent effects of the policy (the details of synthetic control methods are provided in the next section; eFigures 1-4). Fourth, we performed placebo difference-in-differences models by repeating the main analyses with a binary placebo P4P indicator to denote that the HAC-POA policy would be implemented a year before (eTable 6). We also conducted another placebo test by aggregating two years (2006 and 2007) and using a set of those two years as a placebo P4P indicator (the results are not presented). If the placebo P4P policy variables were associated with improvement in surgical care outcomes during placebo years, it would indicate that our results might be due to secular changes in the outcomes. Fifth, we estimated models, adjusted for procedure-specific time trends, to allow for differential time trends between the intervention and control procedures during the pre-policy period (eTable 7).[4] A difference-in-differences model that includes intervention and control procedure-specific time trends allows the pre-existing trends to differ in the two groups, and it can be useful to check the robustness of the results.[13,14] Sixth, we conducted analyses using a different control procedure, including carotid endarterectomy to address concerns related to wound class incomparability between groups (eTables 8). We selected carotid endarterectomy because it is in the same wound class, it is high in volume, and it involves a procedure from a different service line. Finally, we assessed potential cost shifting/decrease in quality in non-complication procedures (eTable 9).

Synthetic control method

We selected the top 100 most performed surgical procedures and excluded procedures that were intervention procedures or similar to the intervention procedures from the synthetic control pool because their inclusion could have reduced the effects. A total of 37 procedures were used in the control pool, including cholecystectomy, appendectomy, abdominal wall repair, small and large intestine surgery, tracheostomy, carotid endarterectomy, urology procedures (e.g., prostatectomy, ureter extirpation), kidney resection, and neurology procedures (e.g., cerebral excision, brain excision). We constructed the synthetic control procedures as a weighted average of available surgical procedures. The weights were chosen to match the outcomes and levels of covariates of the donor surgical procedures to those of the intervention procedures in the pre-policy period. We estimated the weights for SSI and DVT separately to minimize the root mean squared prediction error for each outcome. The covariates and pre-policy outcome that were used to construct the synthetic control procedures for our SSI analysis were: race, gender, age, 29 modified Elixhauser comorbidity index, bed size, ownership, location and teaching status, the natural log of the surgical volume for each hospital, type of admission, and SSI incidence rate for 2005 and 2008. For our DVT analysis, the synthetic control group was constructed with the following variables: bed size, ownership, location, teaching status, type of admission, and DVT incidence rate for 2005 and 2008. The minimum mean squared prediction error was 0.0002 for our SSI model and 0.0001 for our DVT model, indicating an excellent pre-

© 2021 Kim KM et al. *JAMA Network Open.*

policy fit for the synthetic control groups. We assessed statistical significance by conducting a series of placebo tests. We iteratively used a control procedure as if it were the treated procedure from the control pool. From this, we calculated the P value as the proportion of the control procedures that have a ratio of post-intervention mean squared prediction error (MSPE) over pre-intervention MSPE equal to or higher than that of the intervention procedures, standardized by the pre-intervention match quality (i.e., the degree that the control procedures resemble the intervention procedures) (eFigures 3-4). We conducted the analyses using the user-written "synth" and "synth_runner" packages in Stata MP version 15.1 (StataCorp).

© 2021 Kim KM et al. *JAMA Network Open.*

eTable 1. *ICD-9/10-CM* Codes for Defining the Sample

Procedure	ICD-9/10 Procedural Code	Outcome	ICD-9/10 Diagnostic Code
**CMS P4P Target Procedures (P4P Intervention procedures)**			
Cardiac Implantable Electronic Device	00.50, 00.51, 00.52, 00.53, 00.54, 37.80, 37.81, 37.82, 37.83, 37.85, 37.86, 37.87, 37.94, 37.96, 37.98, 37.74, 37.75, 37.76, 37.77, 37.79, 37.89<br><br>02H43JZ, 02H43KZ, 02H43MZ, 02H63JZ, 02H63MZ, 02H73JZ, 02H73MZ, 02HK3JZ, 02HL3JZ, 02HN0JZ, 02HN0MZ, 02HN3JZ, 02HN3MZ, 02HN4JZ, 02HN4MZ, 02PA0MZ, 02PA3MZ, 02PA4MZ,02PAXMZ, 02WA0MZ, 02WA3MZ, 02WA4MZ, 0JH604Z, 0JH605Z, 0JH606Z, 0JH607Z, 0JH608Z, 0JH609Z, 0JH60PZ, 0JH634Z, 0JH635Z, 0JH636Z, 0JH637Z, 0JH638Z, 0JH639Z, 0JH63PZ, 0JH804Z, 0JH805Z, 0JH806Z, 0JH807Z, 0JH808Z, 0JH809Z, 0JH80PZ, 0JH834Z, 0JH835Z, 0JH836Z, 0JH837Z, 0JH838Z, 0JH839Z, 0JH83PZ, 0JPT0PZ, 0JPT3PZ, 0JWT0PZ, 0JWT3PZ	Surgical Site Infection	996.61, 998.59<br><br>K6811, T814XXA, T826XXA, T827XXA
Certain Orthopedic Procedures (Spine/Neck/Shoulder /Ankle)	81.01-81.08, 81.23, 81.24, 81.31-81.38, 81.83, 81.85<br><br>0RG0070, 0RG0071, 0RG007J, 0RG00A0, 0RG00A1, 0RG00AJ, 0RG00J0, 0RG00J1, 0RG00JJ, 0RG00K0, 0RG00K1, 0RG00KJ, 0RG00Z0, 0RG00Z1, 0RG00ZJ, 0RG0370, 0RG0371, 0RG037J, 0RG03A0, 0RG03A1, 0RG03AJ, 0RG03J0, 0RG03J1, 0RG03JJ, 0RG03K0, 0RG03K1, 0RG03KJ, 0RG03Z0, 0RG03Z1, 0RG03ZJ, 0RG0470, 0RG0471, 0RG047J, 0RG04A0, 0RG04A1, 0RG04AJ, 0RG04J0, 0RG04J1, 0RG04JJ, 0RG04K0, 0RG04K1, 0RG04KJ, 0RG04Z0, 0RG04Z1, 0RG04ZJ, 0RG1070, 0RG1071, 0RG107J, 0RG10A0, 0RG10A1, 0RG10AJ, 0RG10J0, 0RG10J1, 0RG10JJ, 0RG10K0, 0RG10K1, 0RG10KJ, 0RG10Z0, 0RG10Z1, 0RG10ZJ, 0RG1370, 0RG1371, 0RG137J, 0RG13A0, 0RG13A1, 0RG13AJ, 0RG13J0, 0RG13J1, 0RG13JJ, 0RG13K0, 0RG13K1, 0RG13KJ, 0RG13Z0, 0RG13Z1, 0RG13ZJ, 0RG1470, 0RG1471, 0RG147J, 0RG14A0, 0RG14A1, 0RG14AJ, 0RG14J0, 0RG14J1, 0RG14JJ, 0RG14K0, 0RG14K1, 0RG14KJ, 0RG14Z0, 0RG14Z1, 0RG14ZJ, 0RG2070, 0RG2071, 0RG207J, 0RG20A0, 0RG20A1, 0RG20AJ, 0RG20J0, 0RG20J1, 0RG20JJ, 0RG20K0, 0RG20K1, 0RG20KJ, 0RG20Z0, 0RG20Z1, 0RG20ZJ, 0RG2370, 0RG2371, 0RG237J, 0RG23A0, 0RG23A1, 0RG23AJ, 0RG23J0, 0RG23J1, 0RG23JJ, 0RG23K0, 0RG23K1, 0RG23KJ, 0RG23Z0, 0RG23Z1, 0RG23ZJ, 0RG2470, 0RG2471, 0RG247J, 0RG24A0, 0RG24A1, 0RG24AJ, 0RG24J0, 0RG24J1, 0RG24JJ, 0RG24K0, 0RG24K1, 0RG24KJ, 0RG24Z0, 0RG24Z1, 0RG24ZJ, 0RG4070, 0RG4071, 0RG407J, 0RG40A0,	Surgical Site Infection	996.67, 998.59<br><br>K6811, T814XXA, T8460XA, T84610A, T84611A<br>T84612A, T84613A, T84614A, T84615A, T84619A, T8463XA, T8469XA, T847XXA

© 2021 Kim KM et al. *JAMA Network Open.*

Procedure	ICD-9/10 Procedural Code	Outcome	ICD-9/10 Diagnostic Code
	0RG40A1, 0RG40AJ, 0RG40J0, 0RG40J1, 0RG40JJ, 0RG40K0, 0RG40K1, 0RG40KJ, 0RG40Z0, 0RG40Z1, 0RG40ZJ, 0RG4370, 0RG4371, 0RG437J, 0RG43A0, 0RG43A1, 0RG43AJ, 0RG43J0, 0RG43J1, 0RG43JJ, 0RG43K0, 0RG43K1, 0RG43KJ, 0RG43Z0, 0RG43Z1, 0RG43ZJ, 0RG4470, 0RG4471, 0RG447J, 0RG44A0, 0RG44A1, 0RG44AJ, 0RG44J0, 0RG44J1, 0RG44JJ, 0RG44K0, 0RG44K1, 0RG44KJ, 0RG44Z0, 0RG44Z1, 0RG44ZJ, 0RG6070, 0RG6071, 0RG607J, 0RG60A0, 0RG60A1, 0RG60AJ, 0RG60J0, 0RG60J1, 0RG60JJ, 0RG60K0, 0RG60K1, 0RG60KJ, 0RG60Z0, 0RG60Z1, 0RG60ZJ, 0RG6370, 0RG6371, 0RG637J, 0RG63A0, 0RG63A1, 0RG63AJ, 0RG63J0, 0RG63J1, 0RG63JJ, 0RG63K0, 0RG63K1, 0RG63KJ, 0RG63Z0, 0RG63Z1, 0RG63ZJ, 0RG6470, 0RG6471, 0RG647J, 0RG64A0, 0RG64A1, 0RG64AJ, 0RG64J0, 0RG64J1, 0RG64JJ, 0RG64K0, 0RG64K1, 0RG64KJ, 0RG64Z0, 0RG64Z1, 0RG64ZJ, 0RG7070, 0RG7071, 0RG707J, 0RG70A0, 0RG70A1, 0RG70AJ, 0RG70J0, 0RG70J1, 0RG70JJ, 0RG70K0, 0RG70K1, 0RG70KJ, 0RG70Z0, 0RG70Z1, 0RG70ZJ, 0RG7370, 0RG7371, 0RG737J, 0RG73A0, 0RG73A1, 0RG73AJ, 0RG73J0, 0RG73J1, 0RG73JJ, 0RG73K0, 0RG73K1, 0RG73KJ, 0RG73Z0, 0RG73Z1, 0RG73ZJ, 0RG7470, 0RG7471, 0RG747J, 0RG74A0, 0RG74A1, 0RG74AJ, 0RG74J0, 0RG74J1, 0RG74JJ, 0RG74K0, 0RG74K1, 0RG74KJ, 0RG74Z0, 0RG74Z1, 0RG74ZJ, 0RG8070, 0RG8071, 0RG807J, 0RG80A0, 0RG80A1, 0RG80AJ, 0RG80J0, 0RG80J1, 0RG80JJ, 0RG80K0, 0RG80K1, 0RG80KJ, 0RG80Z0, 0RG80Z1, 0RG80ZJ, 0RG8370, 0RG8371, 0RG837J, 0RG83A0, 0RG83A1, 0RG83AJ, 0RG83J0, 0RG83J1, 0RG83JJ, 0RG83K0, 0RG83K1, 0RG83KJ, 0RG83Z0, 0RG83Z1, 0RG83ZJ, 0RG8470, 0RG8471, 0RG847J, 0RG84A0, 0RG84A1, 0RG84AJ, 0RG84J0, 0RG84J1, 0RG84JJ, 0RG84K0, 0RG84K1, 0RG84KJ, 0RG84Z0, 0RG84Z1, 0RG84ZJ, 0RGA070, 0RGA071, 0RGA07J, 0RGA0A0, 0RGA0A1, 0RGA0AJ, 0RGA0J0, 0RGA0J1, 0RGA0JJ, 0RGA0K0, 0RGA0K1, 0RGA0KJ, 0RGA0Z0, 0RGA0Z1, 0RGA0ZJ, 0RGA370, 0RGA371, 0RGA37J, 0RGA3A0, 0RGA3A1, 0RGA3AJ, 0RGA3J0, 0RGA3J1, 0RGA3JJ, 0RGA3K0, 0RGA3K1, 0RGA3KJ, 0RGA3Z0, 0RGA3Z1, 0RGA3ZJ, 0RGA470, 0RGA471, 0RGA47J, 0RGA4A0, 0RGA4A1, 0RGA4AJ, 0RGA4J0, 0RGA4J1, 0RGA4JJ, 0RGA4K0, 0RGA4K1, 0RGA4KJ, 0RGA4Z0, 0RGA4Z1, 0RGA4ZJ, 0SG0070, 0SG0071, 0SG007J, 0SG00A0, 0SG00A1, 0SG00AJ, 0SG00J0, 0SG00J1, 0SG00JJ, 0SG00K0, 0SG00K1, 0SG00KJ, 0SG00Z0, 0SG00Z1,		

© 2021 Kim KM et al. *JAMA Network Open.*

Procedure	ICD-9/10 Procedural Code	Outcome	ICD-9/10 Diagnostic Code
	0SG00ZJ, 0SG0370, 0SG0371, 0SG037J, 0SG03A0, 0SG03A1, 0SG03AJ, 0SG03J0, 0SG03J1, 0SG03JJ, 0SG03K0, 0SG03K1, 0SG03KJ, 0SG03Z0, 0SG03Z1, 0SG03ZJ, 0SG0470, 0SG0471, 0SG047J, 0SG04A0, 0SG04A1, 0SG04AJ, 0SG04J0, 0SG04J1, 0SG04JJ, 0SG04K0, 0SG04K1, 0SG04KJ, 0SG04Z0, 0SG04Z1, 0SG04ZJ, 0SG1070, 0SG1071, 0SG107J, 0SG10A0, 0SG10A1, 0SG10AJ, 0SG10J0, 0SG10J1, 0SG10JJ, 0SG10K0, 0SG10K1, 0SG10KJ, 0SG10Z0, 0SG10Z1, 0SG10ZJ, 0SG1370, 0SG1371, 0SG137J, 0SG13A0, 0SG13A1, 0SG13AJ, 0SG13J0, 0SG13J1, 0SG13JJ, 0SG13K0, 0SG13K1, 0SG13KJ, 0SG13Z0, 0SG13Z1, 0SG13ZJ, 0SG1470, 0SG1471, 0SG147J, 0SG14A0, 0SG14A1, 0SG14AJ, 0SG14J0, 0SG14J1, 0SG14JJ, 0SG14K0, 0SG14K1, 0SG14KJ, 0SG14Z0, 0SG14Z1, 0SG14ZJ, 0SG3070, 0SG3071, 0SG307J, 0SG30A0, 0SG30A1, 0SG30AJ, 0SG30J0, 0SG30J1, 0SG30JJ, 0SG30K0, 0SG30K1, 0SG30KJ, 0SG30Z0, 0SG30Z1, 0SG30ZJ, 0SG3370, 0SG3371, 0SG337J, 0SG33A0, 0SG33A1, 0SG33AJ, 0SG33J0, 0SG33J1, 0SG33JJ, 0SG33K0, 0SG33K1, 0SG33KJ, 0SG33Z0, 0SG33Z1, 0SG33ZJ, 0SG3470, 0SG3471, 0SG347J, 0SG34A0, 0SG34A1, 0SG34AJ, 0SG34J0, 0SG34J1, 0SG34JJ, 0SG34K0, 0SG34K1, 0SG34KJ, 0SG34Z0, 0SG34Z1, 0SG34ZJ, 0SG704Z, 0SG707Z, 0SG70JZ, 0SG70KZ, 0SG70ZZ, 0SG734Z, 0SG737Z, 0SG73JZ, 0SG73KZ, 0SG73ZZ, 0SG744Z, 0SG747Z, 0SG74JZ, 0SG74KZ, 0SG74ZZ, 0SG804Z, 0SG807Z, 0SG80JZ, 0SG80KZ, 0SG80ZZ, 0SG834Z, 0SG837Z, 0SG83JZ, 0SG83KZ, 0SG83ZZ, 0SG844Z, 0SG847Z, 0SG84JZ, 0SG84KZ, 0SG84ZZ, XRG0092, XRG1092, XRG2092, XRG4092, XRG6092, XRG7092, XRG8092, XRGA092, XRGB092, XRGC092, XRGD092, 0RGE04Z, 0RGE07Z, 0RGE0JZ, 0RGE0KZ, 0RGE0ZZ, 0RGE34Z, 0RGE37Z, 0RGE3JZ, 0RGE3KZ, 0RGE3ZZ, 0RGE44Z, 0RGE47Z, 0RGE4JZ, 0RGE4KZ, 0RGE4ZZ, 0RGF04Z, 0RGF07Z, 0RGF0JZ, 0RGF0KZ, 0RGF0ZZ, 0RGF34Z, 0RGF37Z, 0RGF3JZ, 0RGF3KZ, 0RGF3ZZ, 0RGF44Z, 0RGF47Z, 0RGF4JZ, 0RGF4KZ, 0RGF4ZZ, 0RGG04Z, 0RGG07Z, 0RGG0JZ, 0RGG0KZ, 0RGG0ZZ, 0RGG34Z, 0RGG37Z, 0RGG3JZ, 0RGG3KZ, 0RGG3ZZ, 0RGG44Z, 0RGG47Z, 0RGG4JZ, 0RGG4KZ, 0RGG4ZZ, 0RGH04Z, 0RGH07Z, 0RGH0JZ, 0RGH0KZ, 0RGH0ZZ, 0RGH34Z, 0RGH37Z, 0RGH3JZ, 0RGH3KZ, 0RGH3ZZ, 0RGH44Z, 0RGH47Z, 0RGH4JZ, 0RGH4KZ, 0RGH4ZZ, 0RGJ04Z, 0RGJ07Z, 0RGJ0JZ, 0RGJ0KZ, 0RGJ0ZZ, 0RGJ34Z, 0RGJ37Z, 0RGJ3JZ, 0RGJ3KZ, 0RGJ3ZZ, 0RGJ44Z, 0RGJ47Z, 0RGJ4JZ, 0RGJ4KZ, 0RGJ4ZZ, 0RGK04Z, 0RGK07Z, 0RGK0JZ, 0RGK0KZ, 0RGK0ZZ, 0RGK34Z, 0RGK37Z, 0RGK3JZ,		

© 2021 Kim KM et al. *JAMA Network Open.*

Procedure	ICD-9/10 Procedural Code	Outcome	ICD-9/10 Diagnostic Code
	0RGK3KZ, 0RGK3ZZ, 0RGK44Z, 0RGK47Z, 0RGK4JZ, 0RGK4KZ, 0RGK4ZZ, 0RQE0ZZ, 0RQE3ZZ, 0RQE4ZZ, 0RQEXZZ, 0RQF0ZZ, 0RQF3ZZ, 0RQF4ZZ, 0RQFXZZ, 0RQG0ZZ, 0RQG3ZZ, 0RQG4ZZ, 0RQGXZZ, 0RQH0ZZ, 0RQH3ZZ, 0RQH4ZZ, 0RQHXZZ, 0RQJ0ZZ, 0RQJ3ZZ, 0RQJ4ZZ, 0RQJXZZ, 0RQK0ZZ, 0RQK3ZZ, 0RQK4ZZ, 0RQKXZZ, 0RUE07Z, 0RUE0JZ, 0RUE0KZ, 0RUE37Z, 0RUE3JZ, 0RUE3KZ, 0RUE47Z, 0RUE4JZ, 0RUE4KZ, 0RUF07Z, 0RUF0JZ, 0RUF0KZ, 0RUF37Z, 0RUF3JZ, 0RUF3KZ, 0RUF47Z, 0RUF4JZ, 0RUF4KZ, 0RUG07Z, 0RUG0JZ, 0RUG0KZ, 0RUG37Z, 0RUG3JZ, 0RUG3KZ, 0RUG47Z, 0RUG4JZ, 0RUG4KZ, 0RUH07Z, 0RUH0JZ, 0RUH0KZ, 0RUH37Z, 0RUH3JZ, 0RUH3KZ, 0RUH47Z, 0RUH4JZ, 0RUH4KZ, 0RUJ07Z, 0RUJ0JZ, 0RUJ0KZ, 0RUJ37Z, 0RUJ3JZ, 0RUJ3KZ, 0RUJ47Z, 0RUJ4JZ, 0RUJ4KZ, 0RUK07Z, 0RUK0JZ, 0RUK0KZ, 0RUK37Z, 0RUK3JZ, 0RUK3KZ, 0RUK47Z, 0RUK4JZ, 0RUK4KZ, 0RGL04Z, 0RGL05Z, 0RGL07Z, 0RGL0JZ, 0RGL0KZ, 0RGL0ZZ, 0RGL34Z, 0RGL35Z, 0RGL37Z, 0RGL3JZ, 0RGL3KZ, 0RGL3ZZ, 0RGL44Z, 0RGL45Z, 0RGL47Z, 0RGL4JZ, 0RGL4KZ, 0RGL4ZZ, 0RGM04Z, 0RGM05Z, 0RGM07Z, 0RGM0JZ, 0RGM0KZ, 0RGM0ZZ, 0RGM34Z, 0RGM35Z, 0RGM37Z, 0RGM3JZ, 0RGM3KZ, 0RGM3ZZ, 0RGM44Z, 0RGM45Z, 0RGM47Z, 0RGM4JZ, 0RGM4KZ, 0RGM4ZZ, 0RQL0ZZ, 0RQL3ZZ, 0RQL4ZZ, 0RQLXZZ, 0RQM0ZZ, 0RQM3ZZ, 0RQM4ZZ, 0RQMXZZ, 0RUL07Z, 0RUL0JZ, 0RUL0KZ, 0RUL37Z, 0RUL3JZ, 0RUL3KZ, 0RUL47Z, 0RUL4JZ, 0RUL4KZ, 0RUM07Z, 0RUM0JZ, 0RUM0KZ, 0RUM37Z, 0RUM3JZ, 0RUM3KZ, 0RUM47Z, 0RUM4JZ, 0RUM4KZ		
Bariatric Surgery for Obesity	44.38, 44.39, 44.95<br><br>0D16079, 0D1607A, 0D1607B, 0D1607L, 0D160J9, 0D160JA, 0D160JB, 0D160JL, 0D160K9, 0D160KA, 0D160KB, 0D160KL, 0D160Z9, 0D160ZA, 0D160ZB, 0D160ZL, 0D16479, 0D1647A, 0D1647B, 0D1647L, 0D164J9, 0D164JA, 0D164JB, 0D164JL, 0D164K9, 0D164KA, 0D164KB, 0D164KL, 0D164Z9, 0D164ZA, 0D164ZB, 0D164ZL, 0D16879, 0D1687A, 0D1687B, 0D1687L, 0D168J9, 0D168JA, 0D168JB, 0D168JL, 0D168K9, 0D168KA, 0D168KB, 0D168KL, 0D168Z9, 0D168ZA, 0D168ZB, 0D168ZL, L0DV64CZ	Surgical Site Infection	278.01 & (one of the following: 539.01, 539.81, 998.59)<br><br>E6601 & one of the following: K6811, K9501, K9581, T814XXA)
Certain Orthopedic Procedures	00.85-00.87, 81.51-81.52, or 81.54	Deep Vein Thrombosis	415.11, 415.13, 415.19, 453.40-453.42

© 2021 Kim KM et al. *JAMA Network Open.*

Procedure	ICD-9/10 Procedural Code	Outcome	ICD-9/10 Diagnostic Code
(Total Knee Arthroplasty /Total Hip Arthroplasty)	0SR9019, 0SR901A, 0SR901Z, 0SR9029, 0SR902A, 0SR902Z, 0SR9039, 0SR903A, 0SR903Z, 0SR9049, 0SR904A, 0SR904Z, 0SR907Z, 0SR90J9, 0SR90JA, 0SR90JZ, 0SR90KZ, 0SRA009, 0SRA00A, 0SRA00Z, 0SRA019, 0SRA01A, 0SRA01Z, 0SRA039, 0SRA03A, 0SRA03Z,0SRA07Z, 0SRA0J9, 0SRA0JA, 0SRA0JZ, 0SRA0KZ, 0SRB019,0SRB01A, 0SRB01Z, 0SRB029, 0SRB02A, 0SRB02Z, 0SRB039, 0SRB03A, 0SRB03Z, 0SRB049, 0SRB04A, 0SRB04Z, 0SRB07Z, 0SRB0J9, 0SRB0JA, 0SRB0JZ, 0SRB0KZ, 0SRC07Z, 0SRC0J9, 0SRC0JA, 0SRC0JZ, 0SRC0KZ, 0SRC0L9, 0SRC0LA, 0SRC0LZ,0SRD07Z, 0SRD0J9, 0SRD0JA, 0SRD0JZ, 0SRD0KZ, 0SRD0L9, 0SRD0LA, 0SRD0LZ, 0SRE009, 0SRE00A, 0SRE00Z, 0SRE019, 0SRE01A, 0SRE01Z, 0SRE039, 0SRE03A, 0SRE03Z, 0SRE07Z, 0SRE0J9, 0SRE0JA, 0SRE0JZ, 0SRE0KZ, 0SRR019, 0SRR01A, 0SRR01Z, 0SRR039, 0SRR03A, 0SRR03Z, 0SRR07Z, 0SRR0J9, 0SRR0JA, 0SRR0JZ, 0SRR0KZ, 0SRS019, 0SRS01A, 0SRS01Z, 0SRS039, 0SRS03A, 0SRS03Z, 0SRS07Z, 0SRS0J9, 0SRS0JA, 0SRS0JZ, 0SRS0KZ, 0SRT07Z, 0SRT0J9, 0SRT0JA, 0SRT0JZ, 0SRT0KZ, 0SRU07Z, 0SRU0J9, 0SRU0JA, 0SRU0JZ, 0SRU0KZ, 0SRV07Z, 0SRV0J9, 0SRV0JA, 0SRV0JZ, 0SRV0KZ, 0SRW07Z, 0SRW0J9, 0SRW0JA, 0SRW0JZ, 0SRW0KZ, 0SU90BZ, 0SUA0BZ, 0SUB0BZ, 0SUE0BZ, 0SUR0BZ, 0SUS0BZ		I2602, I2609, I2692, I2699, I82401, I82402, I82403, I82409, I82411, I82412, I82413, I82419, I82421, I82422, I82423, I82429, I82431, I82432, I82433, I82439, I82441, I82442, I82443, I82449, I82491, I82492, I82493, I82499, I824Y1, I824Y2, I824Y3, I824Y9, I824Z1, I824Z2, I824Z3, I824Z9
**CMS P4P Non-Target Procedures (P4P Control procedures)**			
Laparoscopic Cholecystectomy	51.23, 51.24<br><br>0FB44ZX, 0FB44ZZ, 0FB48ZX, 0FB48ZZ, 0FT44ZZ	Surgical Site Infection	567, 567.2, 567.21, 567.22, 567.23, 567.29, 567.3, 567.38, 567.39, 567.8, 567.81, 567.89, 567.9, 682.2<br><br>T8140XA, T8140XD, T8140XS, T8141XA, T8141XD, T8141XS, T8142XA, T8142XD, T8142XS, T8143XA, T8143XD, T8143XS, T8144XA, T8144XD, T8144XS, T8149XA, T8149XD, T8149XS
		Deep Vein Thrombosis	415.11, 415.13, 415.19, 453.40-453.42

© 2021 Kim KM et al. *JAMA Network Open.*

Procedure	ICD-9/10 Procedural Code	Outcome	ICD-9/10 Diagnostic Code
			I2602, I2609, I2692, I2699, I82401, I82402, I82403, I82409, I82411, I82412, I82413, I82419, I82421, I82422, I82423, I82429, I82431, I82432, I82433, I82439, I82441, I82442, I82443, I82449, I82491, I82492, I82493, I82499, I824Y1, I824Y2, I824Y3, I824Y9, I824Z1, I824Z2, I824Z3, I824Z9
Laparoscopic Appendectomy	47.01<br><br>0DBJ4ZX, 0DBJ4ZZ, 0DBJ8ZX, 0DBJ8ZZ, 0DTJ4ZZ, 0DTJ8ZZ	Surgical Site Infection	567, 567.2, 567.21, 567.22, 567.23, 567.29, 567.3, 567.38, 567.39, 567.8, 567.81, 567.89, 567.9, 682.2<br><br>T8140XA, T8140XD, T8140XS, T8141XA, T8141XD, T8141XS, T8142XA, T8142XD, T8142XS, T8143XA, T8143XD, T8143XS, T8144XA, T8144XD, T8144XS, T8149XA, T8149XD, T8149XS
		Deep Vein Thrombosis	415.11, 415.13, 415.19, 453.40-453.42<br><br>I2602, I2609, I2692, I2699, I82401, I82402, I82403, I82409, I82411, I82412, I82413, I82419, I82421, I82422, I82423, I82429, I82431, I82432, I82433, I82439,

© 2021 Kim KM et al. *JAMA Network Open.*

Procedure	ICD-9/10 Procedural Code	Outcome	ICD-9/10 Diagnostic Code
			I82441, I82442, I82443, I82449, I82491, I82492, I82493, I82499, I824Y1, I824Y2, I824Y3, I824Y9, I824Z1, I824Z2, I824Z3, I824Z9
**Control Procedures for Sensitivity Analysis**			
Carotid Endarterectomy	433.10<br>38.02, 03CK0ZZ, 03CL0ZZ	Surgical Site Infection	857, 998.59<br><br>T8140XA, T8140XD, T8140XS, T8141XA, T8141XD, T8141XS, T8142XA, T8142XD, T8142XS, T8143XA, T8143XD, T8143XS, T8149XA, T8149XD, T8149XS
		Deep Vein Thrombosis	415.11, 415.13, 415.19, 453.40-453.42<br><br>I2602, I2609, I2692, I2699, I82401, I82402, I82403, I82409, I82411, I82412, I82413, I82419, I82421, I82422, I82423, I82429, I82431, I82432, I82433, I82439, I82441, I82442, I82443, I82449, I82491, I82492, I82493, I82499, I824Y1, I824Y2, I824Y3, I824Y9, I824Z1, I824Z2, I824Z3, I824Z9

Abbreviations: ICD-9/10-CM: International Classification of Diseases, Ninth/Tenth, Clinical Modification; CMS: Centers for Medicare and Medicaid; P4P: pay-for-performance

© 2021 Kim KM et al. *JAMA Network Open.*

eTable 2. Surgical Complication Rates for High-Volume Surgical Procedures[a] in the Healthcare Cost and Utilization Project Data FY 2005-2017, grouped by intervention procedures and control procedures, as one of the four criteria to select a control group in a difference-in-differences analysis

Procedures	Mean surgical site infection rates	Mean deep vein thrombosis rates
**Intervention procedures (Targeted by the HAC-POA)**	0.4	0.7
**Control procedures (Non-targeted by the HAC-POA)**		
Laparoscopic appendectomy	0.7	0.4
Laparoscopic cholecystectomy	1.2	0.5
**High-volume procedures considered as control procedures**		
Abdominal wall repair	52.9	22.9
Craniotomy	2.0	2.4
Endoscopic kidney resection	7.1	1.0
Endoscopic treatment of ureteric stone	1.9	1.1
Endovascular repair of abdominal aortic aneurysm	4.4	0.5
Evacuation of subdural hematomas	1.6	2.7
Hernia repair	7.1	1.9
Hysteroscopic uterus resection	6.0	0.4
Large intestine resection	12.3	2.2
Lumbar vertebral disc excision	2.3	0.4
Open aortic valve replacement	12.5	1.1
Open appendectomy	1.9	0.6
Open carotid endarterectomy	3.3	0.1
Open cholecystectomy	4.7	0.9
Open foot excision, subcutaneous and fascia	10.3	1.9
Open large Intestine resection	9.0	1.8
Open reduction internal fixation, Fibular	1.1	0.7
Open reduction internal fixation, Intramedullary nail, Femur	2.6	2.6
Open small intestine excision	9.8	1.9
Open small intestine resection	16.7	2.3
Percutaneous ablation	4.0	0.7
Percutaneous atrium infusion device insertion	1.6	2.5
Percutaneous coronary artery intervention, one artery	3.4	0.4
Percutaneous Inferior vena cava Intraluminal device insertion	2.6	58.8
Percutaneous sigmoid colon excision	8.8	1.9
Prostate resection	3.2	1.2
Release lumbar nerve	9.3	13.8
Total shoulder	1.0	0.2
Tracheostomy	22.3	12.7
Abdominal wall repair	52.9	22.9

© 2021 Kim KM et al. *JAMA Network Open.*

[a] We used four criteria to select the control procedures, including procedures that have similar complication rates on average. This table shows the average surgical complication rates of intervention, control, and other high-volume procedures.

© 2021 Kim KM et al. *JAMA Network Open.*

eTable 3. Propensity Score-Unweighted[a] Characteristics of Hospital Stays[a] for the Pre-Policy Intervention Group[b], the Post-Policy Intervention Group, the Pre-Policy Control Group[c], and the Post-Policy Control Group

					Weighted Standardized Difference in Means		
	I Pre	I Post	C Pre	C Post	I Post vs. I Pre	C Pre vs. I Pre	C Post vs. I Pre
Gender, %							
Male	39.4	37.8	43.9	47.5	-0.03	0.09	0.16
Female	60.6	62.2	56.1	52.5	0.03	-0.09	-0.16
Race/ethnicity, %							
White	88.7	87.2	81.8	79.1	-0.05	-0.19	-0.26
Black	4.2	5.3	5.1	6.4	0.05	0.04	0.10
Hispanic	3.8	4.0	7.8	9.3	0.01	0.17	0.22
Asian/Pacific Islander	1.2	1.2	2.7	2.6	-0.00	0.10	0.10
Others	2.1	2.3	2.6	2.6	0.01	0.03	0.03
Age, mean (SD), years	75.5 (7.0)	74.6 (6.9)	75.5 (7.1)	75.7 (7.3)	-0.14	0.00	0.03
Median Income Quartile, %							
0-25th percentile	22.8	22.3	25.3	25.7	-0.01	0.06	0.07
26-50th percentile	27.2	26.5	25.6	25.9	-0.02	-0.04	-0.03
51-75th percentile	26.0	26.5	25.2	25.6	0.01	-0.02	-0.01
76-100th percentile	23.9	24.7	23.8	22.8	0.02	-0.00	-0.03
Elixhauser Comorbidity Index, %							
Acquired Immune Deficiency syndrome	0.0	0.0	0.0	0.0	0.01	0.01	0.02
Alcohol abuse	0.7	1.0	0.9	1.7	0.04	0.03	0.09
Anemia	13.9	13.2	10.1	15.8	-0.02	-0.12	0.05
Rheumatoid arthritis /Collagen Vascular Disease	3.1	4.2	2.3	2.9	0.06	-0.05	-0.01
Chronic blood loss anemia	1.5	1.3	0.5	0.6	-0.02	-0.10	-0.08
Congestive heart failure	4.6	5.0	9.3	11.6	0.02	0.19	0.26
Chronic pulmonary disease	15.1	16.1	17.7	18.5	0.03	0.7	0.09
Coagulopathy	1.8	3.2	2.1	4.9	0.09	0.02	0.18
Depression	7.4	11.7	5.6	8.1	0.15	-0.06	0.02
Diabetes, uncomplicated	18.6	19.3	22.0	25.6	0.02	0.09	0.17
Diabetes with chronic complications	1.5	3.1	2.1	3.7	0.10	0.04	0.13
Drug abuse	0.1	0.3	0.1	0.3	0.05	0.00	0.05
Hypertension	68.0	72.5	66.9	73.7	0.10	-0.02	0.13
Hypothyroidism	15.3	18.5	13.1	15.9	0.09	-0.06	0.02
Liver disease	0.5	0.8	2.9	4.7	0.05	0.19	0.27

© 2021 Kim KM et al. *JAMA Network Open.*

Lymphoma	0.4	0.4	0.6	0.8	0.01	0.03	0.05
Fluid and electrolyte disorders	10.2	12.4	20.0	29.1	0.07	0.27	0.49
Metastatic cancer	0.4	0.4	1.2	1.4	0.00	0.09	0.11
Neurological disorders	5.3	6.4	5.0	6.2	0.05	-0.01	0.04
Obesity	7.6	15.0	7.0	12.7	0.23	-0.02	0.17
Paralysis	0.8	0.8	1.3	1.4	0.01	0.04	0.06
Peripheral vascular disorders	3.8	3.9	5.5	7.1	0.01	0.08	0.15
Psychoses	1.1	1.5	1.2	1.8	0.04	0.00	0.06
Pulmonary circulation disorders	1.0	1.4	1.3	2.7	0.04	0.04	0.13
Renal failure	4.4	8.5	6.0	12.7	0.17	0.07	0.30
Solid tumor without metastasis	0.9	0.8	1.5	1.8	-0.01	0.05	0.08
Peptic ulcer disease excluding bleeding	0.0	0.1	0.1	0.1	0.03	0.03	0.02
Valvular disease	5.0	5.2	6.6	7.2	0.01	0.07	0.09
Weight loss	0.7	1.2	2.3	5.4	0.06	0.13	0.28
Location and Teaching Status, %							
Rural	7.3	8.8	11.7	10.7	0.05	0.14	0.12
Urban, non teaching	53.0	39.4	57.3	48.5	-0.27	0.09	-0.09
Urban, teaching	39.6	51.8	31.0	40.8	0.25	-0.18	0.02
Ownership, %							
Government	47.3	8.7	44.0	10.0	-0.95	-0.07	-0.91
Private	52.7	91.3	56.0	90.0	0.95	0.07	0.91
Bed Size, %							
Small	12.6	19.5	10.3	11.9	0.19	-0.07	-0.12
Medium	23.0	26.5	24.8	27.8	0.08	0.04	0.11
Large	64.5	54.0	65.0	60.3	-0.21	0.01	-0.08

Abbreviations: I = Intervention procedures; C = Control procedures; Pre = Pre-policy period (before the third quarter of 2008); Post = Post-policy period (after the third quarter of 2008)

[a] Characteristics are not propensity-score weighted, but survey weights are accounted for. Rows may not add up to 100%, due to rounding.
[b] An intervention group includes patients who underwent the HAC-POA policy's targeted procedures (cardiac [implantable electronic devices], orthopedic [spine, neck, shoulder, elbow, total knee, and total hip replacement], and obesity-related bariatric procedures). A control group includes patients who underwent an appendectomy and a cholecystectomy.

© 2021 Kim KM et al. *JAMA Network Open.*

eTable 4. Covariate Unadjusted Propensity Score-Weighted Surgical Complication Rates, Length of Stay, and Hospital Costs Before and After the HAC-POA Program Implementation for Hospital Stays for Intervention Procedures[a] and Control Procedures[b]

Outcome	Pre Policy Period (2005-2008)	Post Policy Period (2009-2017)	Unadjusted Difference-in-Differences Estimate[c] (95% CI)
Surgical Complications, No. of Events/ No. of Patients (%)			
SSI			
Intervention Procedures	169/44 584 (0.38)	660/182 427 (0.35)	-0.33 (-0.50 to -0.15)
Control Procedures	219/29 487 (0.66)	1180/89 110 (0.93)	
DVT			
Intervention Procedures	1078/149 492 (0.72)	5222/822 059 (0.58)	0.02 (-0.11 to 0.16)
Control Procedures	131/29 487 (0.45)	419/89 110 (0.25)	
Mortality No. of Events/ No. of Patients (%)			
Intervention Procedures	681/194 076 (0.35)	3900/1 004 589 (0.38)	-0.04 (-0.14 to 0.06)
Control Procedures	167/29 487 (0.35)	738/89 110 (0.39)	
Length of Stay, mean (SD), days			
Intervention Procedures	4.0 (2.50)	3.7 (4.95)	-0.65 (-0.72 to -0.58)
Control Procedures	4.1 (1.17)	4.3 (2.04)	
Hospital Costs, mean (SD),[d] $			
Intervention Procedures	22 938.4 (13 066.9)	21 584.5 (26 349.0)	- 2655.9 (-2845.7 to -2466.0)
Control Procedures	13 684.7 (3244.7)	14343.7 (5212.5)	

Abbreviations: HAC-POA, Hospital Acquired Conditions-Present On Admission; SSI, Surgical Site Infection; DVT, Deep Vein Thrombosis; CI, Confidence Interval

[a] Intervention procedures include patients who underwent the HAC-POA policy's targeted procedures (cardiac [implantable electronic devices], orthopedic [spine, neck, shoulder, elbow], and obesity-related bariatric procedures for SSI; total knee and total hip replacements for DVT; and all of the stated procedures for length of stay, mortality, and hospital costs).
[b] Control procedures include patients who underwent laparoscopic appendectomy and laparoscopic cholecystectomy.
[c] The unadjusted difference-in-differences estimate is a propensity score-weighted unadjusted differential effect of the policy between the intervention procedures and control procedures before and after the policy implementation. Estimates for SSI, DVT, and mortality are predicted probability changes in percentage points. Estimates for length of stay are changes in days.

© 2021 Kim KM et al. *JAMA Network Open.*

Estimates for hospital costs measured for an inpatient stay with cost-to-charge ratios, are changes in percentage.

[d] Adjusted for inflation using the personal consumption expenditures health-by-function index for the 2017 dollar value.

© 2021 Kim KM et al. *JAMA Network Open.*

eTable 5. Propensity Score-Weighted Estimates[a] of the Association between the HAC-POA Program and Surgical Site Infection, Deep Vein Thrombosis, and Mortality from the Logistic Regression Model[b] and Hospital Costs from a Generalized Linear Model[c]

Outcome	Difference in the Intervention Procedures[d] (95% CI)	Difference in the Control Procedures[e] (95% CI)	Difference-in-Differences Estimate (95% CI)
SSI	-1.21 (-2.36 to -0.07)	-0.16 (-0.30 to -0.02)	-0.12 (-0.25 to -0.00)
DVT	-0.15 (-0.34 to 0.04)	-0.20 (-0.40 to 0.00)	0.04 (-0.16 to 0.26)
Mortality	-0.11 (-0.26 to 0.04)	-0.13 (-0.30 to 0.04)	0.02 (-0.08 to 0.13)
Hospital Costs	-3756.84 (05389.91 to -2123.76)	-872.97 (-1125.01 to -620.92)	-2726.33 (-3562.37 to -1890.30)

Abbreviations: HAC-POA, Hospital Acquired Conditions-Present On Admission; SSI, Surgical Site Infection; DVT, Deep Vein Thrombosis; CI, Confidence Interval

[a] Estimates are predicted probability changes in percentage points.
[b] Logistic regression was used to model surgical site infection, deep vein thrombosis, and mortality.
[c] A one-part generalized linear model with gamma distribution and log link was used to model hospital costs.
[d] The difference in the intervention procedures is the difference in the average marginal effect of the outcome between the pre- and post-policy implementation period for patients in the intervention procedures (i.e., cardiac [implantable electronic devices], orthopedic [spine, neck, shoulder, elbow, total knee, and total hip replacement], and obesity-related bariatric procedures).
[d] The difference in the control procedures is the difference in the average marginal effect of the outcome between the pre- and post-policy implementation period among patients in the control procedures (i.e., laparoscopic cholecystectomy and laparoscopic appendectomy).

© 2021 Kim KM et al. *JAMA Network Open.*

eTable 6. Propensity Score-Weighted Estimates[a] of the Association between the HAC-POA Program and Surgical Outcomes from Difference-in-Differences Models Using a Placebo Implementation Year[b]

Outcome	Difference in the Intervention Procedures[c] (95% CI)	Difference in the Control Procedures[d] (95% CI)	Difference-in-Differences Estimate (95% CI)
SSI	0.35 (0.06 to 0.65)	0.20 (-0.18 to 0.58)	0.15 (-0.21 to 0.53)
DVT	0.08 (-0.14 to 0.31)	0.27 (-0.16 to 0.70)	-0.18 (-0.50 to 0.14)
Length of Stay	-0.11 (-0.21 to -0.02)	-0.06 (-0.20 to 0.08)	-0.05 (-0.19 to 0.08)
Mortality	-0.03 (-0.13 to 0.08)	-0.09 (-0.29 to 0.11)	0.06 (-0.10 to 0.22)
Hospital Costs	-3.77 (-6.36 to -1.19)	-3.34 (-5.81 to -0.87)	-0.44 (-338 to 2.51)

Abbreviations: HAC-POA, Hospital Acquired Conditions-Present On Admission; SSI, Surgical Site Infection; DVT, Deep Vein Thrombosis; CI, Confidence Interval

[a] Estimates for SSI, DVT, and mortality are changes in percentage points. Estimates for length of stay are changes in days. Estimates for hospital costs are changes in percentage.
[b]We performed placebo difference-in-differences models by repeating the main analyses with a binary placebo P4P indicator to denote that the HAC-POA policy would be implemented a year before.
[c] The difference in the intervention procedures is the difference in the average marginal effect of the outcome between the pre- and post-policy implementation period for patients in the intervention procedures (i.e., cardiac [implantable electronic devices], orthopedic [spine, neck, shoulder, elbow], and obesity-related bariatric procedures for SSI; total knee and total hip replacement for DVT; and all of the stated procedures for length of stay, mortality, and hospital costs).
[d] The difference in the control procedures is the difference in the average marginal effect of the outcome between the pre- and post-policy implementation period among patients in the control procedures (i.e., laparoscopic cholecystectomy and laparoscopic appendectomy).

© 2021 Kim KM et al. *JAMA Network Open.*

eTable 7. Propensity Score-Weighted Estimates[a] of the Association between the HAC-POA Program and Surgical Outcomes from Models using Intervention and Control Group-Specific Pre-Intervention Time Trends

Outcome	Difference in the Intervention Proceduresb (95% CI)	Difference in the Control Proceduresc (95% CI)	Difference-in-Differences Estimate (95% CI)
SSI	0.07 (-0.27 to 0.41)	0.34 (-0.06 to 0.73)	-0.27 (-0.64 to -0.10)
DVT	-0.00 (-0.20 to 0.20)	-0.06 (-0.28 to 0.16)	0.06 (-0.16 to 0.28)
Length of Stay	-0.60 (-0.72 to -0.49)	0.38 (0.26 to 0.51)	-0.99 (-1.14 to -0.83)
Mortality	0.17 (0.06 to 0.28)	-0.02 (-0.21 to 0.17)	0.19 (-0.01 to 0.37)
Hospital Costs	-3.44 (-6.34 to -0.55)	9.38 (6.29 to 12.47)	-12.82 (-15.88 to -9.76)

Abbreviations: HAC-POA, Hospital Acquired Conditions-Present On Admission; SSI, Surgical Site Infection; DVT, Deep Vein Thrombosis; CI, Confidence Interval

[a] Estimates for SSI, DVT, and mortality are changes in percentage points. Estimates for length of stay are changes in days. Estimates for hospital costs are changes in percentage.
[b] The difference in the intervention procedures is the difference in the average marginal effect of the outcome between the pre- and post-policy implementation period for patients in the intervention procedures (i.e., cardiac [implantable electronic devices], orthopedic [spine, neck, shoulder, elbow], and obesity-related bariatric procedures for SSI; total knee and total hip replacement for DVT; and all of the stated procedures for length of stay, mortality, and hospital costs).
[c] The difference in the control procedures is the difference in the average marginal effect of the outcome between the pre- and post-policy implementation period among patients in the control procedures (i.e., laparoscopic cholecystectomy and laparoscopic appendectomy).

© 2021 Kim KM et al. *JAMA Network Open.*

eTable 8. Propensity Score-Weighted Estimates[a] of the Association between the HAC-POA Program and Surgical Outcomes among Patients who Underwent the Target Surgical Procedures and Patients who Underwent Carotid Endarterectomy

Outcome	Difference in the Intervention Procedures[b] (95% CI)	Difference in the Control Procedures[c] (95% CI)	Difference-in-Differences Estimate (95% CI)
SSI	-0.51 (-1.09 to 0.06)	0.27 (-0.27 to 0.83)	-0.79 (-1.22 to -0.37)
DVT	0.03 (-0.16 to 0.22)	0.17 (-0.02 to 0.37)	-0.14 (-0.31 to 0.03)
Length of Stay	-0.93 (-1.09 to -0.77)	-0.31 (-0.48 to -0.13)	-0.62 (-0.75 to -0.50)
Mortality	0.17 (-0.10 to 0.44)	0.56 (0.27 to 0.86)	-0.40 (-0.65 to 0.15)
Hospital Costs	11.30 (6.48 to 16.12)	25.74 (20.3 to 31.2)	-14.43 (-17.88 to -11.00)

[a] Estimates for SSI, DVT, and mortality are changes in percentage points. Estimates for length of stay are changes in days. Estimates for hospital costs are changes in percentage.
[b] The difference in the intervention procedures is the difference in the average marginal effect of the outcome between the pre- and post-policy implementation period for Medicare patients who underwent the targeted procedures of the HAC-POA policy.
[b] The difference in the control procedures is the difference in the average marginal effect of the outcome between the pre- and post-policy implementation period among Medicare patients who underwent carotid endarterectomy.

© 2021 Kim KM et al. *JAMA Network Open.*

eTable 9. Propensity Score-Weighted Estimates of the Association between the HAC-POA Program and Surgical Outcomes in Patients with Surgical Complications[a] vs. Patients without Surgical Complications

Outcome	Difference in the Intervention Proceduresb (95% CI)		Difference in the Control Proceduresc (95% CI)		Difference-in-Difference-in-Differences Estimated (95% CI)
	Pre- and Post- policy changes among patients Without surgical complication[e] (95% CI)	Pre- and Post-policy changes among patients With surgical complication[f] (95% CI)	Pre- and Post- policy changes among patient Without surgical complicatio[g] (95% CI)	Pre- and Post-policy changes among patient With surgical complication[h] (95% CI)	
Length of Stay	-1.25 (-1.39 to -1.12)	-2.20 (-2.72to -1.67)	-0.73 (-0.86 to -0.60)	-0.62 (-1.59 to 0.35)	-1.05 (-2.12 to 0.01)
Mortality	0.08 (-0.09 to 0.25)	-0.50 (-1.96 to 0.97)	0.04 (-0.14 to 0.21)	-1.52 (-4.05 to 1.01)	0.98 (-1.88 to 3.85)
Hospital Costs	-2.58 (-6.47 to 1.31)	-6.14 (-11.322 to -0.96)	5.46 (1.65 to 9.28)	7.93 (-3.15 to 19.02)	-6.03 (-17.29 to 5.22)

[a] Surgical complications are defined as any of SSI or DVT.
[b] Intervention procedures include patients who underwent the HAC-POA policy's targeted procedures (cardiac [implantable electronic devices], orthopedic [spine, neck, shoulder, elbow, total knee, and total hip replacement], and obesity-related bariatric procedures).
[c] Control procedures include patients who underwent laparoscopic appendectomy and laparoscopic cholecystectomy.
[d] The difference-in-difference-in-differences estimate is a differential effect of the policy between the intervention and the control procedures before and after the policy implementation (difference-in-differences) across patients with surgical complications vs. no complications. Estimates for length of stay are changes in days. Estimates for mortality are predicted probability changes in percentage points. Estimates for hospital costs are changes in percentage.
[e] The difference in the intervention procedures without surgical complications is the difference in the average marginal effect of the outcome in the intervention procedures between pre- and post-policy implementation among patients without surgical complications (SSI or DVT).
[f] The difference in the intervention procedures with surgical complications is the difference in the average marginal effect of the outcome in the intervention procedures between pre- and post-policy implementation among patients with surgical complications.
[g] The difference in the control procedures without surgical complications is the difference in the

© 2021 Kim KM et al. *JAMA Network Open.*

average marginal effect of the outcome in the control procedures between pre- and post-policy implementation among patients without surgical complications.

[h] The difference in the control procedures with surgical complications is the difference in the average marginal effect of the outcome in the control procedures between pre- and post-policy implementation among patients with surgical complications.

© 2021 Kim KM et al. *JAMA Network Open.*

eFigure 1. Estimates of the Association between the HAC-POA Program and Surgical Site Infections: Comparison Between the Intervention Procedures and the Synthetic Control Procedures[a]

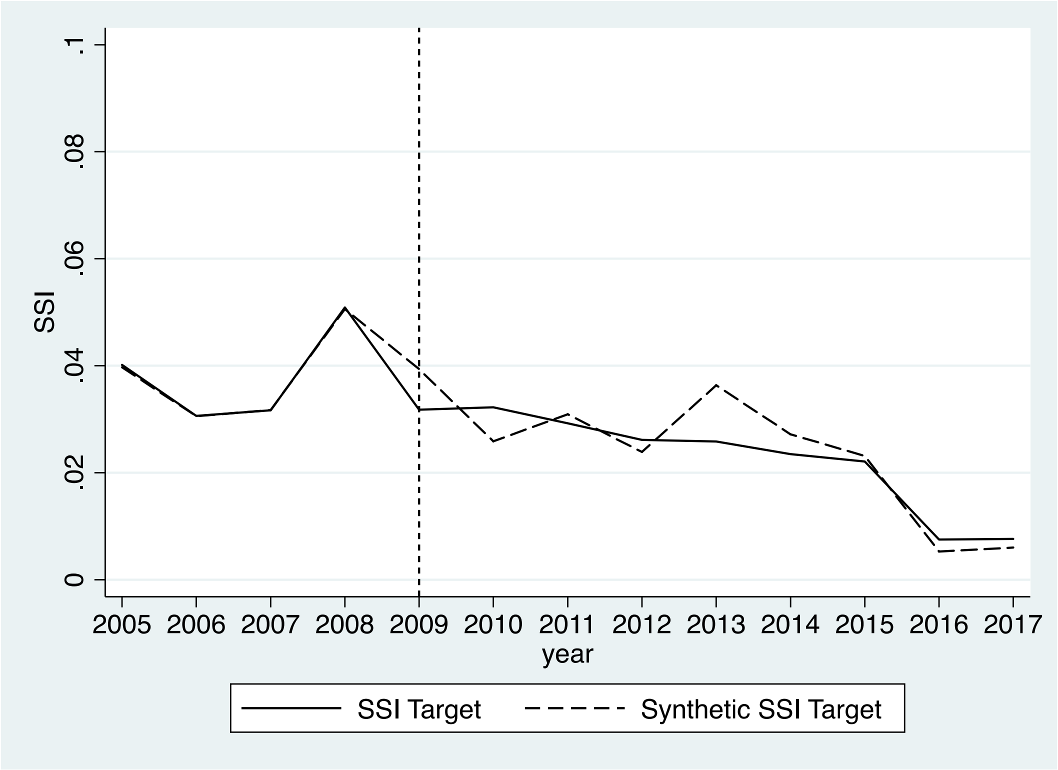

Abbreviations: HAC-POA, Hospital Acquired Conditions-Present On Admission
[a] Details about the synthetic control method are provided in eStudy Methods. A total of 37 procedures were used in the control pool, including cholecystectomy, appendectomy, abdominal wall repair, small and large intestine surgery, tracheostomy, carotid endarterectomy, urology procedures (e.g., prostatectomy, ureter extirpation), kidney resection, and neurology procedures (e.g., cerebral excision, brain excision).

© 2021 Kim KM et al. *JAMA Network Open.*

eFigure 2. Estimates of the Association between the HAC-POA Program and Deep Vein Thrombosis: Comparison Between the Target Procedures and the Synthetic Control Procedures[a]

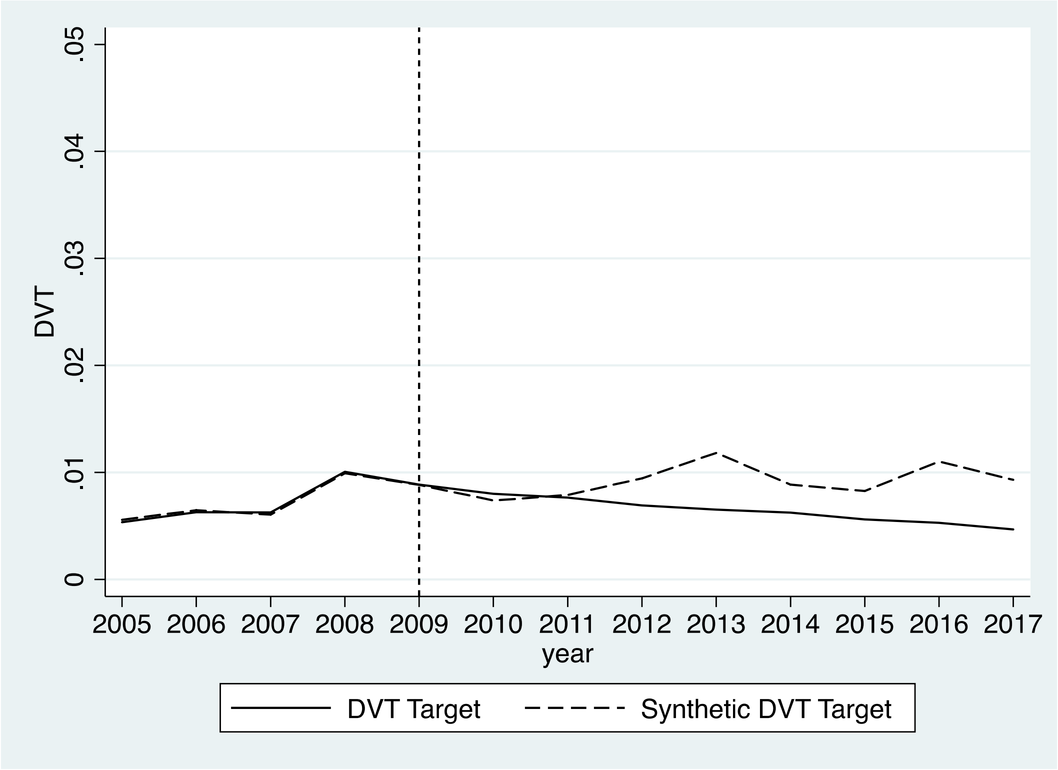

Abbreviations: HAC-POA, Hospital Acquired Conditions-Present On Admission
[a] Details about the synthetic control method are provided in eStudy Methods. A total of 37 procedures were used in the control pool, including cholecystectomy, appendectomy, abdominal wall repair, small and large intestine surgery, tracheostomy, carotid endarterectomy, urology procedures (e.g., prostatectomy, ureter extirpation), kidney resection, and neurology procedures (e.g., cerebral excision, brain excision).

© 2021 Kim KM et al. *JAMA Network Open.*

eFigure 3. Ratio of post-policy MSPE and pre-policy MSPE[a]: Surgical Site Infection

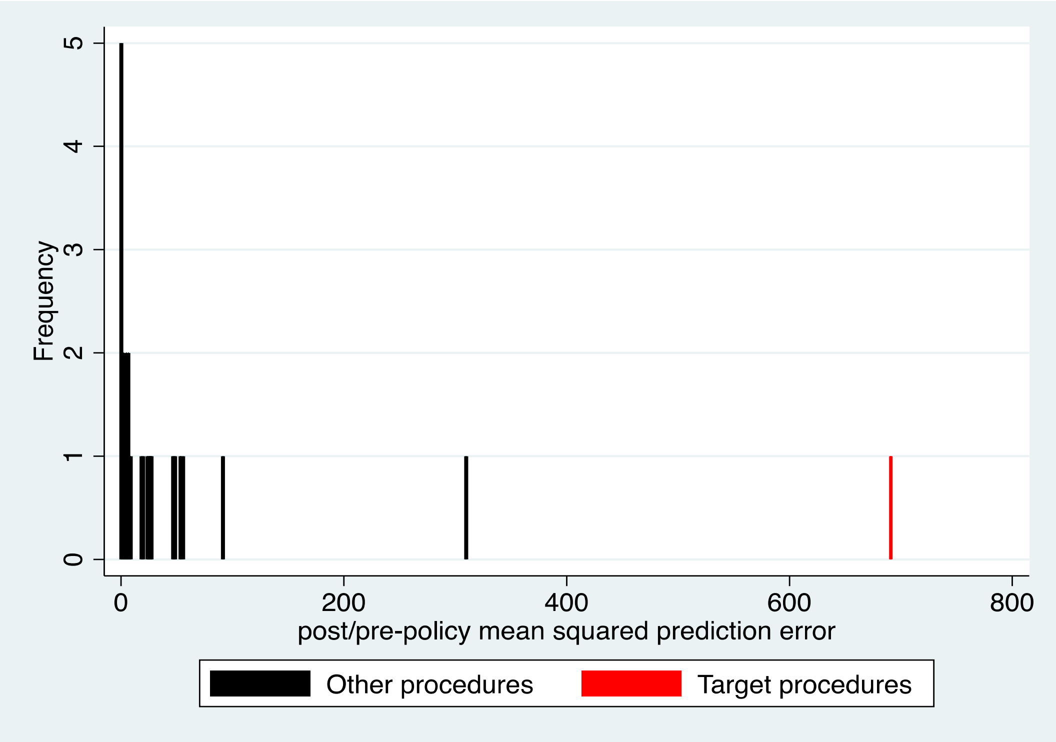

Abbreviation: MSPE, Mean squared prediction error
[a]The distribution of the ratios of post-policy MSPE and pre-policy MSPE indicates whether the results from the placebo tests using the synthetic control procedures in the pool could have occurred by chance. If the placebo effects are equally large as the intervention group effects, it is likely that the estimated effect was observed by chance. The ratio for the intervention procedures is about 690 times the MSPE for the pre-policy period and there was no control procedure achieved such a large ratio, which indicates the observed intervention effect was unlikely to be by chance.

© 2021 Kim KM et al. *JAMA Network Open.*

eFigure 4. Ratio of post-policy MSPE and pre-policy MSPE[a]: Deep Vein Thrombosis

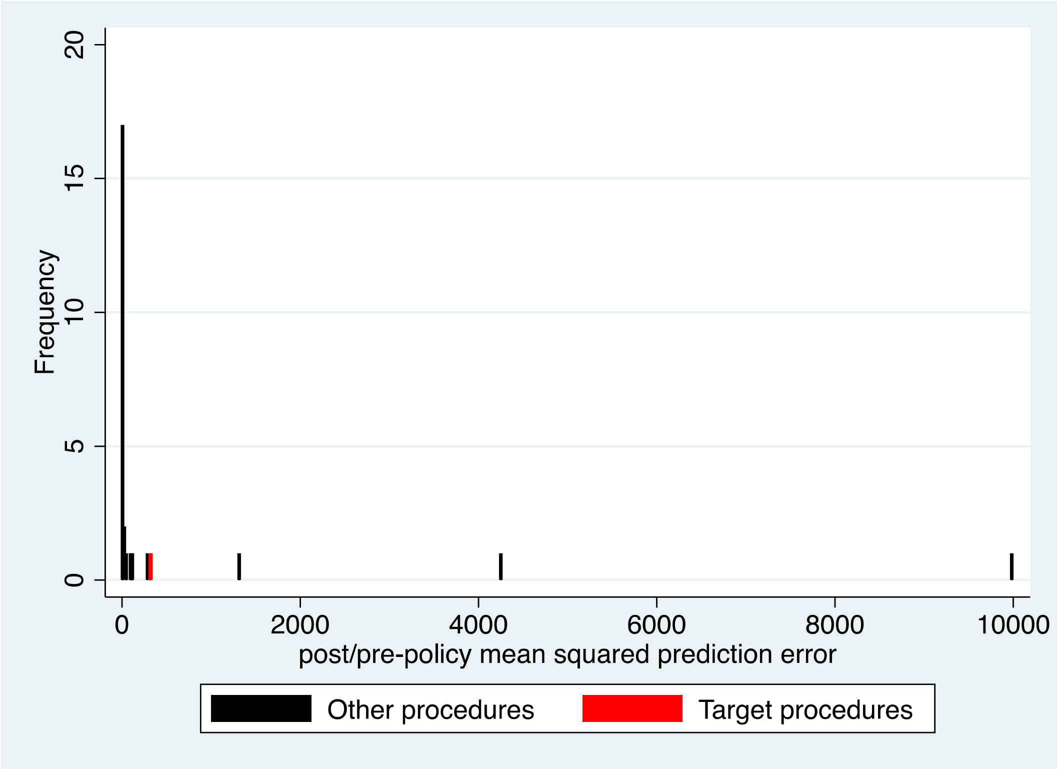

Abbreviations: MSPE, Mean squared prediction error

[a] The ratio of post/pre-intervention MSPE is larger in some of the placebo runs. The distribution of the ratios of post/pre-intervention MSPE indicates that the estimated effect that the policy had on the incidence of deep vein thrombosis may be observed by chance.

© 2021 Kim KM et al. *JAMA Network Open.*

eReferences

1. Ryan AM. Effects of the premier hospital quality incentive demonstration on Medicare patient mortality and cost. *Health Services Research*. 2009;44(3):821-842. doi:10.1111/j.1475-6773.2009.00956.x
2. Kwong JZ, Weng Y, Finnegan M, et al. Effect of Medicare's nonpayment policy on surgical site infections following orthopedic procedures. *Infection Control & Hospital Epidemiology*. 2017;38(7):817-822. doi:10.1017/ice.2017.86
3. Gidwani R, Bhattacharya J. CMS reimbursement reform and the incidence of hospital-acquired pulmonary embolism or deep vein thrombosis. *J GEN INTERN MED*. 2015;30(5):588-596. doi:10.1007/s11606-014-3087-3
4. Ryan AM, Burgess JF, Dimick JB. Why we should not be indifferent to specification choices for difference-in-differences. *Health Services Research*. 2015;50(4):1211-1235. doi:10.1111/1475-6773.12270
5. Healy D, Cromwell J. *Hospital-Acquired Conditions – Present on Admission: Examination of Spillover Effects and Unintended Consequences*. Centers for Medicare and Medicaid Services; 2012. https://www.cms.gov/medicare/medicare-fee-for-service-payment/hospitalacqcond/downloads/hac-spillovereffects.pdf
6. Cerulli G. *Econometric Evaluation of Socio-Economic Programs*. Vol 49. Springer Berlin Heidelberg; 2015. doi:10.1007/978-3-662-46405-2
7. Imbens GW, Wooldridge JM. Lecture notes 10: difference-in-differences estimation. Published online July 31, 2007. http://www.nber.org/WNE/lect_10_diffindiffs.pdf
8. Stuart EA, Huskamp HA, Duckworth K, et al. Using propensity scores in difference-in-differences models to estimate the effects of a policy change. *Health Services and Outcomes Research Methodology*. 2014;14(4):166-182.
9. Stuart EA. Matching methods for causal inference: a review and a look forward. *Statistical Science*. 2010;25(1):1-21. doi:10.1214/09-STS313
10. Rosenbaum PR, Rubin DB. Constructing a control group using multivariate matched sampling methods that incorporate the propensity score. *The American Statistician*. 1985;39(1):33-38. doi:10.1080/00031305.1985.10479383
11. Deb P, Norton EC, Manning WG. *Health Econometrics Using Stata*. 1st ed. Stata Press; 2017.
12. Manning WG, Mullahy J. Estimating log models: to transform or not to transform? *Journal of Health Economics*. 2001;20(4):461-494.
13. Angrist JD, Pischke J-S. *Mastering Metrics: The Path from Cause to Effect*. Princeton University Press; 2015.
14. Besley T, Burgess R. Can labor regulation hinder economic performance? Evidence from India. *The Quarterly Journal of Economics*. 2004;119(1):91-134. doi:10.1162/003355304772839533

© 2021 Kim KM et al. *JAMA Network Open.*
